# Modeling Memory Effects in a Head-Final Language With Category Locality

**DOI:** 10.1162/OPMI.a.384

**Published:** 2026-08-21

**Authors:** Shinnosuke Isono, Kohei Kajikawa, Yohei Oseki, Masayuki Asahara

**Affiliations:** National Institute for Japanese Language and Linguistics (NINJAL), Tokyo, Japan; Georgetown University, Washington, DC, USA; University of Tokyo, Tokyo, Japan

**Keywords:** psycholinguistics, sentence comprehension, memory, Japanese, Combinatory Categorial Grammar

## Abstract

Memory limitations have been assumed to be a major factor shaping human sentence comprehension, but characterizing memory-demanding structures in a broad-coverage and cross-linguistic manner has remained a challenge. A recent study suggested that such a general characterization can be obtained by assuming efficient compression of the syntactic information from the input guided by a logic-based grammar. Specifically, it was shown that the memory cost of syntactic integration measured in terms of Combinatory Categorial Grammar (CCG) accounts for variance in reading times for naturalistic texts in English, unlike the more traditional dependency-based integration cost. The crucial test for the new theory is yet to be done, however, since the predictions of CCG- and dependency-based integration cost most drastically diverge in head-final languages. This study conducts this crucial test using a naturalistic reading time dataset in Japanese. Our results favor CCG over dependency grammar: CCG-based integration cost predicted reading time variance of held-out data, while the dependency-based version failed to show such a predictive power. Our results, combined with previous ones, suggest that the logic-based grammar captures some cross-linguistic, potentially universal aspect of human sentence comprehension.

## INTRODUCTION

Understanding a written or spoken sentence requires reconstruction of the structured meaning from the linear sequence of words that is perceived. How this is achieved is a central question in the study of human sentence comprehension. Many theories of comprehension have assumed that it involves encoding and retrieval of information using working memory (Demberg et al., 2013; Gibson, 2000; Isono, 2024; Lewis & Vasishth, 2005; McElree, 2000). These theories point to various factors that can tax working memory and make sentences difficult to comprehend.

One possible structural factor is distance, or *locality*. A widely-held assumption in linguistics and cognitive science more generally is that information encoded in working memory degrades gradually. This is either because of time-based decay, interference with subsequent information, or both (Anderson et al., 2004; Lewis & Vasishth, 2005; Oberauer et al., 2012; Ricker et al., 2016; Van Dyke & McElree, 2011). As representations degrade, they become harder to retrieve and integrate with later information. Applied to sentence comprehension, this view predicts that linguistic elements that are related to each other but linearly distant in presentation will be difficult to process. This processing difficulty is called a *locality effect*.

A simple and popular approach to predict locality effects is to use a dependency grammar, as proposed by *Dependency Locality Theory* (DLT) (Gibson, 1998, 2000; see also Futrell, Gibson, & Levy, 2020). In (1), for example, *reporter* has to be retrieved from working memory when *disliked* is encountered downstream to establish the subject-verb relation between them. Since this dependency is longer in (1b) than in (1a), DLT predicts that *disliked* is more difficult to process in (1b). The difficulty is expected to be observed as an increase in the time spent on the word in spontaneous reading, for example.

(1) a. The reporter who the senator attacked disliked the editor.
    b. The reporter who the senator who John met attacked disliked the editor.

Evidence for such locality effects, however, has been mixed. Robust effects have been found in certain constructions such as English relative clauses (Bartek et al., 2011; Grodner & Gibson, 2005; Roland et al., 2021; Staub, 2010), but are absent in others such as verb-final structures of German and Japanese (Levy & Keller, 2013; Nakatani & Gibson, 2010). The effect is also missing in large-scale reading time data based on naturalistic corpora in English (Demberg & Keller, 2008; Demberg et al., 2013; Dotlačil, 2021). This instability of locality effects contrasts with expectation-based effects (Hale, 2001; Levy, 2008) (i.e., less expected input is more difficult), which have gained much empirical support across languages and constructions (e.g., Wilcox et al., 2023).

Addressing these problems, Isono (2024) recently proposed a new theory of locality effect based on Combinatory Categorial Grammar (CCG) (Steedman, 1996, 2000), dubbed *Category Locality Theory* (CLT). According to this work, earlier theories of locality effect faced empirical problems not because locality is an insignificant factor in human sentence comprehension but because of the way the distance is measured. Isono argued that syntactic distance measured in terms of CCG rather than dependencies explains the seemingly puzzling previous results, specifically the processing difficulty in English relative clauses and the lack of difficulty in verb-final structures. Furthermore, the study demonstrated that the CCG-based distance predicts reading times for an English naturalistic corpus (Natural Stories corpus, Futrell et al. (2021)), even when expectation-based predictors are controlled for. These results suggest that CCG reflects some important aspects of the syntactic representation used by the human parser.

With this background, this study further tests the prediction of CLT and compares it with that of DLT using naturalistic texts in Japanese. More precisely, we ask the following questions:

- Do DLT and CLT explain variance in reading times in Japanese naturalistic reading?
- If both do, which explains more? Does one explain variance that the other does not?

The language is typologically different from English as it is strictly head-final, and is notorious for not following the dependency-based prediction of locality effects (Nakatani, 2021; Nakatani & Gibson, 2010). While Isono (2024) claims that CLT accounts for the lack of the locality effect at clause-final verbs, whether CLT predicts reading times in verb-final languages in a broad-coverage manner has not been investigated empirically. To anticipate the results, our results favor CLT over DLT.

The rest of this paper is structured as follows. The Background section introduces CCG and CLT, and compares CLT’s qualitative predictions with those based on dependency grammar. The Corpus-Based Experiment section reports the results of the corpus analysis. The Discussion section discusses the implications of the results.

## BACKGROUND

### Combinatory Categorial Grammar

This subsection introduces Combinatory Categorial Grammar (CCG) (Ades & Steedman, 1982; Steedman, 1996, 2000), which is the basis of the theory tested in this study, CLT. A key characteristic of CCG is that the structure-building mechanism for the competence-level derivation can also be used to model incremental performance. CLT is concerned with the cost of those structure-building operations. We therefore begin by introducing CCG as a competence grammar and then describe how they are used to model performance.

CCG (Steedman, 1996, 2000) belongs to a family of grammar formalisms called *categorial grammars* (Ajdukiewicz, 1935/1967; Bar-Hillel, 1953; Kubota & Levine, 2020; Lambek, 1958, 1961; Moortgat, 1997; Morrill, 1994, i.a.). In categorial grammars, including CCG, each linguistic expression is associated with a category that specifies its syntactic behavior. The set of categories is defined inductively as follows:

(2) a. There is a finite set of primitive categories: *S*, *NP*, *N*, ….
    b. If *X* and *Y* are (primitive or functional) categories, *X*/*Y* and *X*\*Y* are functional categories.^1^

The basic behavior of functional categories is specified by the *application* rules, defined below.

(3) 
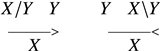

*X* and *Y* stand for an arbitrary category. In each rule, the categories above the line are the input categories (or premises), and the one below the line is the output category (or conclusion); beside the line is the symbol for the rule. The following is an example of a simple derivation using just these rules:

(4) 
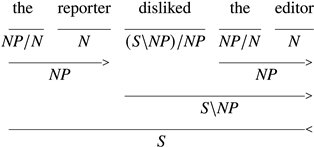

This system resembles feature-based implementations of Merge in Minimalist syntax (Adger, 2003; Chomsky, 1995; Stabler, 2012). Like uninterpretable features in Minimalism, argument categories (/*Y* or \*Y*) specify the features of the expression it combines with, and are consumed once such a combination is made. Categorial grammars differ from Minimalism in that they allow complex categories to be targeted as an argument. For example, the following analysis of a relative clause assumes that the relative pronoun selects a complex category *S*\*NP*, effectively binding the missing subject:

(5) 
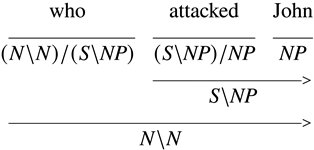

Categorial grammar just with the application rules, called AB grammar after Ajdukiewicz (1935/1967) and Bar-Hillel (1953), is not expressive enough to analyze natural languages. Various extensions have been proposed to overcome its limitations (de Groote, 2001; Kubota & Levine, 2020; Lambek, 1958, 1961; Moortgat, 1997; Morrill, 1994; Morrill et al., 2011; Muskens, 2003, i.a.). CCG is one of such extensions, and characteristically uses combinatory rules, which are associated with combinators from the combinatory logic (Curry & Feys, 1958). While the exact set of combinatory rules available is debatable (Hoyt & Baldridge, 2008), the most important ones are the (harmonic, first-order) composition rules (**B**) and type-raising rules (**T**)^2^, defined below:

(6) 
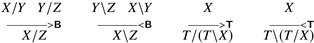

Here, *T* stands for a variable category. When there are multiple variable categories in the same derivation, they will be distinguished by an index (*T*_1_, *T*_2_, …). As the name suggests, composition rules compose two functional categories into a new functional category. Type-raising rules modify an argument category to a functional category. The following analysis of an object-extracted relative clause uses these rules:

(7) 
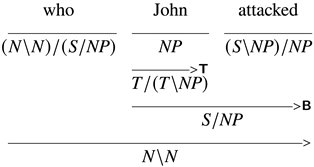

Note that in this analysis, the string *John attacked* is analyzed as having the category *S*/*NP*. This is an appropriate characterization of the string, since it would be a full sentence if it is followed by an object NP. As can be seen from this example, the combination of composition and type-raising is powerful enough to model basic displacement phenomena.

It is important to bear in mind that CCG derivations and CCG constituents are conceptually different from derivations and constituents in the mainstream generative grammar. CCG derivations like (7) produce a tree-like structure, but the tree is not a representation. Rather, it is a record of the computational process. Rules never refer to the structure of the tree and only use the most recent result at the root of the tree. CCG constituents are also very different from conventional constituents. Any string can form a constituent if some CCG rules can assign a category to it, including unsaturated functional categories like *S*/*NP*.

### Incremental Processing With CCG

In (7), the category *S*/*NP* assigned to *John attacked* is understood as describing the string as a gapped clause. Another interpretation of the same category is that it describes the state of the parser that has processed *John attacked* and awaits an incoming NP. Thus, the combinatory rules in CCG, used to account for competence-level data such as relative clauses, can also be used to model incremental processing. The incremental processing of a sentence, say *John attacked Bill*, can be illustrated as a derivation, as shown below.

(8) 
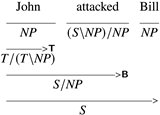

This derivation is said to be *incremental* because it integrates each word from left to right into a single constituent. This contrasts with the normal form tree (Eisner, 1996), which makes the least use of composition:

(9) 
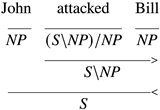

In CCG (and in categorial grammars in general), there is a transparent mapping between syntax and semantics via the Curry-Howard correspondence. Having a syntactic analysis for a fragment therefore guarantees that there is a logical form reflecting the syntactic structure. In the current case, the *S*/*NP* category for *John attacked* is associated with the term *λx*.**attacked**(*x*)(**John**), appropriately expressing that John is the agent of the attacking action and the patient term is waited for (for details about compositional semantics in CCG, see Steedman, 2000, 2019). This accounts for the well-known fact that human incremental sentence processing has immediate access to the semantics of the fragments as well as their syntax (Kamide et al., 2003; Tanenhaus et al., 1995).

### Category Locality Theory

Since CCG is compatible with incremental parsing, several studies have used it to model behavioral and neural data during language processing (Kajikawa et al., 2024; Ozaki et al., 2024; Stanojević et al., 2023). While these studies use the number of rule applications as the predictor, *Category Locality Theory* (CLT) (Isono, 2024) proposed to use CCG to model memory constraints. The key feature of CCG that is important for CLT is that it does not provide a fully incremental parse for every sentence (Demberg, 2012). One of the structures that cannot be parsed fully incrementally with the canonical rules mentioned above is the object relative clause. (10) is the parse that is as incremental as possible:

(10) 
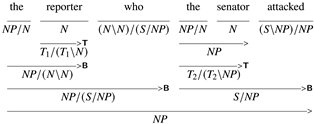

Here the embedded subject *the senator* is not combined directly with the preceding *the reporter who* because there is no applicable rule that leads to an appropriate parse:

(11) 
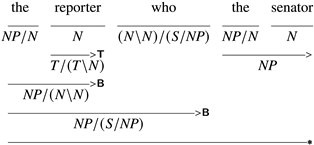

Therefore, at the time *the senator* is processed, the parser has to maintain two separate categories/constituents. The core idea of CLT is that having separate CCG constituents as in the current example taxes working memory. The original paper discusses two ways in which working memory is taxed. In the current paper we will refer to these ways as *integration cost* and *storage cost* respectively, following the terminology of DLT (Gibson, 2000).

The integration cost models the locality effect, and is incurred when an old constituent is retrieved from memory for integration with a newer constituent. The cost should be larger when the distance between the point of the formation of the constituent and the point of its retrieval is longer. This prediction is derived from two widely-held assumptions in psycholinguistics (Demberg et al., 2013; Gibson, 2000; Lewis & Vasishth, 2005; McElree, 2000). First, the activation of a linguistic representation held in working memory degrades over time, through time-based decay, interference, or both. Second, the latency of the retrieval of a representation is dependent on its activation. The exact forms of the activation decay and the activation–retrieval latency relation are not part of the core claim of CLT; Isono (2024) distinguishes those as “auxiliary assumptions” to implement CLT. In this paper we will empirically test several possible measures of distance.

In the current relative clause example, an integration cost is predicted at *attacked*, where *the reporter who* is retrieved for integration with *the senator attacked*. The cost is determined by the length of the intervening material, *the senator* in this case. Isono (2024) argues that CLT integration cost accounts for the well-known processing difficulty of English object-relative clauses (Bartek et al., 2011; Grodner & Gibson, 2005; King & Just, 1991; Roland et al., 2021; Staub, 2010). Isono also shows that it is a significant predictor of reading times for English texts in the Natural Stories corpus (Futrell et al., 2021). DLT integration cost (Gibson, 2000; Shain et al., 2016), in contrast, showed no significant effect for the same dataset.

Isono (2024) also considers the storage cost of CCG-based parsing. This idea is borrowed from DLT (Gibson, 2000). DLT assumes that the storage of the structure built so far can slow down processing of new materials since the two processes use the same pool of cognitive resources. The storage cost is quantified by the number of syntactic heads required to complete the sentence. Controlled experiments in English and Spanish support the presence of DLT storage cost (Chen et al., 2005; Ristic et al., 2022). In CCG-based parsing, the storage cost should be contingent on the number of separate constituents, which have to be held in working memory for later integration. In the current case, the storage of *the reporter who* should slow down the processing of *the senator*. Isono (2024) shows that CCG-based storage cost is also a significant predictor of reading times for the Natural Stories corpus. Though the original work does not include CCG-based storage cost in CLT proper (the integration cost was the only component of CLT), this paper will refer to it as CLT storage cost because we then have a nice correspondence between the integration/storage costs in CLT and DLT.

When integration and storage costs arise during the processing of a sentence depends on when CCG fails to provide a fully incremental parse, and when it fails to depends on the set of rules available. In the current discussion we assume most basic rules of application (>, <), first-order harmonic composition (**B**), and type-raising (**T**). But there are other rules discussed in the literature, and they can affect psycholinguistic predictions. For example, adding a rule known as the >**D** rule (Hoyt & Baldridge, 2008) makes the derivation of a English object relative clause (10) more incremental (Demberg, 2012), enabling the integration of *the reporter who* and *the senator* without *attacked*. This would shift the locus of the integration cost to *senator*. An extreme case would be a complete set of combinatory rules that can analyze any left fragment of a grammatical expression as a single constituent. A parser with such a complete set of rules would achieve fully incremental parsing based on powerful prediction, and CLT predicts no locality effects at all. The previous result by Isono (2024) suggests that this is not the case, since reading times for an English corpus could be modeled by a less effortful CCG parser. In fact, reading times for the Natural Stories corpus were better predicted without the >**D** rule. In the corpus analysis of this study, we will also consider different rule sets.

### Comparison With DLT

CLT resembles DLT in that it assumes that non-locality in syntactic computation incurs processing costs. The difference between the two theories lies in the syntactic structure adopted. Generally, CLT predicts non-locality less often because CCG can summarize the previous input into a small number of categories (often one) while dependency grammar maintains each head as an independent chunk.

Figure 1 illustrates the predictions of CLT and DLT for the same object relative clause.^3^ In the figure, arcs above the text represent dependencies. Non-local dependencies, indicated by blue arcs, should incur processing costs: a storage cost is predicted in areas covered by the arc, and an integration cost proportional to the length of the arc is predicted at the right end of the arc. Below the text is the graph for CCG-based processing. Here, each node represents the category that the parser assigned to the string that has been observed so far. The edges indicate how these categories are integrated to yield new categories for larger constituents. Here we illustrate CCG parsing as a system of state transition with multiple states held in parallel when necessary, as in Wanner and Maratsos (1978). When an integration is non-local, as indicated by a red edge, storage and integration costs are predicted. Again, the storage cost should affect the processing of the area covered by the edge, and the integration cost should be incurred at the right end of the edge. As can be seen from the figure, both CLT and DLT predict a non-zero integration cost (i.e., a locality effect) at the embedded verb, and a non-zero storage cost over the embedded subject. These predictions match previous experimental observations, but the two theories posit different sources of the observed locality effect: in DLT, it is the length of the dependencies; in CLT, it is the complexity of the logical/grammatical structure, which prevents fully incremental integration.

**Figure 1.**
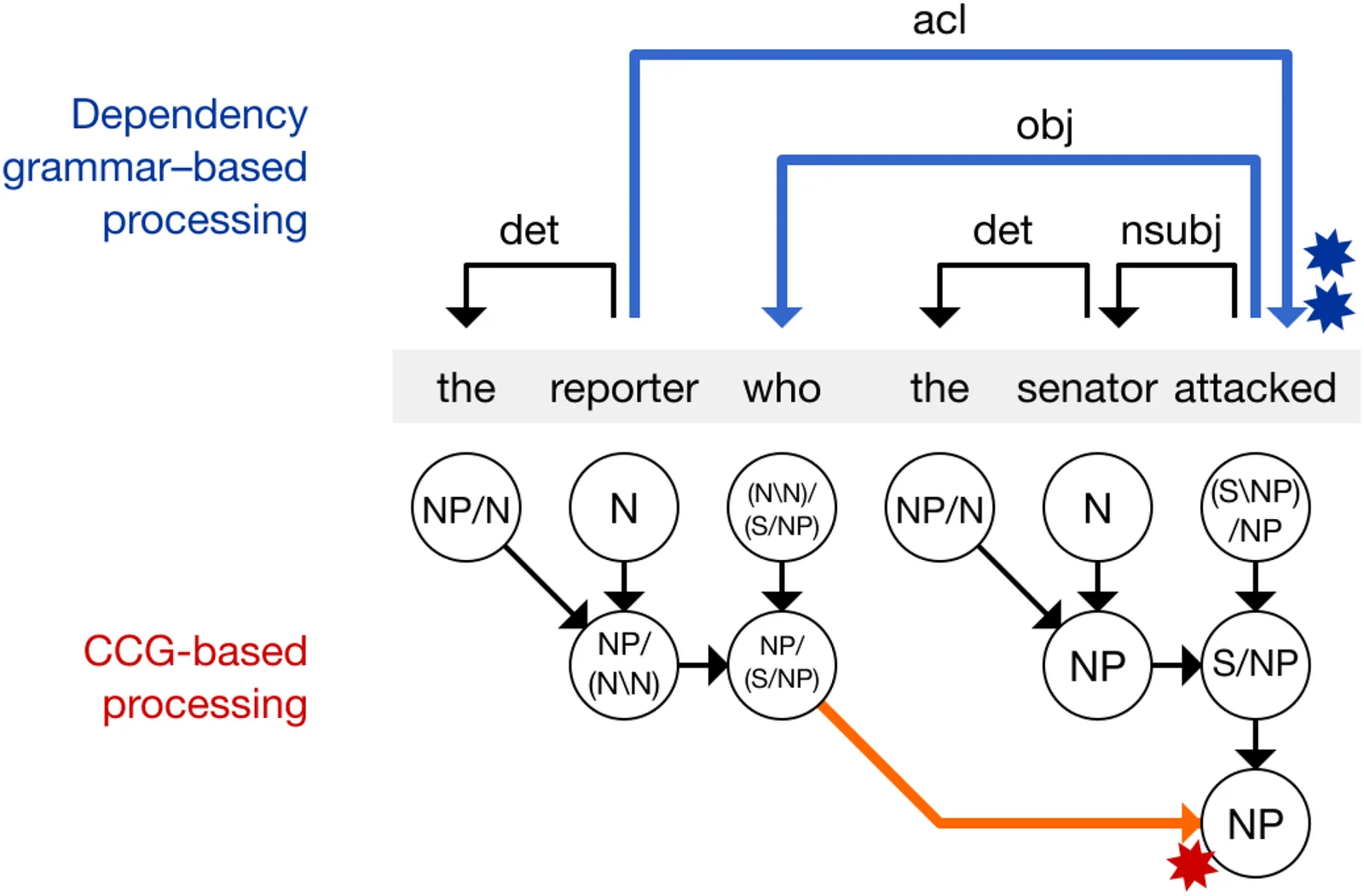
Dependency grammar–based and CCG-based processing of an English object-extracted relative clause. Colored arcs/edges indicate non-local integrations. Octagram stars indicate where a non-zero integration cost is predicted. The regions covered by the colored arcs/edges are where a non-zero storage cost is predicted.

While DLT and CLT make similar predictions regarding English relative clauses, which is important given that the locality effect in this construction is robust, they make different predictions in other constructions. For example, DLT predicts locality effects in English matrix subject-verb dependencies, but CLT does not (controlled experiments on this point show mixed results (Bartek et al., 2011; Grodner & Gibson, 2005)). Another notable example is the verb-final structure, which will be discussed below. As Isono (2024) notes, however, DLT and CLT are not necessarily mutually exclusive accounts of processing cost. Both theories may contribute to the prediction of reading times if different components of our linguistic faculty, such as semantics and syntax, employ dependency- and CCG-like representations, respectively. From this consideration, in our corpus analysis, we test statistical models that include DLT and CLT simultaneously, in addition to models with just one of the theories.

### CLT and Japanese

Isono (2024) also discusses its prediction for verb-final structures in languages like Japanese. Consider the following simple SOV sentence:

(12) Kisya-ga  giin-o  koogekisita.
    reporter-nom senator-acc attacked
    ‘(The) reporter attacked (the) senator.’

In a simple dependency-based processing, two dependencies are completed at the verb: one with the subject and another with the object. DLT therefore predicts a locality effect due to the length of these dependencies. For example, when the subject-verb distance is lengthened by an intervening element, as in (13), the verb should be harder to process than in (12).

(13) a. Kisya-ga  kizi-de giin-o   koogekisita.
    reporter-nom article-in senator-acc attacked
    ‘(The) reporter attacked (the) senator in (the) article.’
    b. Kisya-ga  yotoo-no   giin-o   koogekisita.
    reporter-nom ruling.party-gen senator-acc attacked
    ‘(The) reporter attacked (the) senator of (the) ruling party.’

A factor that must be controlled for to evaluate this prediction is the facilitatory effect of the intervening element. A matrix adverbial like *kizi-de* ‘in (the) article’ in (13a) can have a facilitatory effect on the verb in two ways: by reinforcing the activation of the predicted verb node (Vasishth & Lewis, 2006), and by making the verb more predictable (Hale, 2001; Konieczny, 2000; Levy, 2008). Such a facilitatory effect would cancel out the locality effect. A structure like (13b) is more suitable for the evaluation of the locality effect at the verb because the intervening element is a modifier on a matrix argument, and is therefore less likely to affect the activation or prediction of the verb. Isono and Hirose (2023) report that Japanese subject-verb dependencies still fail to show a locality effect even when structures like (13b) are used (also see Nakatani & Gibson, 2008, 2010). This result is problematic for DLT.

Unlike DLT, CLT predicts no locality effect at clause-final verbs. The normal-form CCG analysis of (13b) is as follows, ignoring morphological details:

(14) 
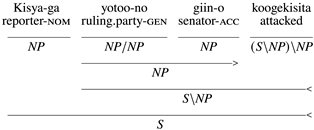

The maximally incremental version is the following:

(15) 
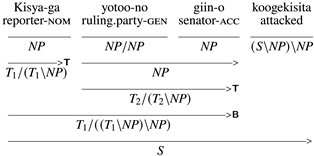

A key property of CCG here is that it allows the pre-verb argument cluster *kisya-ga yotoo-no giin-o* to form a single constituent of category *T*_1_/((*T*_1_\*NP*)\*NP*), correctly characterizing it as expecting a transitive predicate. The integration of the subsequent verb *koogekisita* is local, hence no locality effect.

Notably, it is not the case that CLT predicts a null effect for all regions of a verb-final structure. For example, (15) is not fully incremental because the subject *kisya-ga* and the object modifier *yotoo-no* cannot be combined. This predicts a locality effect at the object head *giin-o*. Such locality effects between clausemates in Japanese are observed in self-paced reading experiments reported by Isono and Hirose (2023). See Figure 3 in the Experiment section for a visual illustration of the difference between the predictions of DLT and CLT for a verb-final structure. This is an example of the general divergence of DLT and CLT predictions in head-final structures: DLT predicts integration cost at the final head, while CLT predicts integration cost at dependents before the head.

One observation that still needs explanation is that Japanese clause-final verbs do show a locality effect when the argument is a *wh*-element (Ono & Nakatani, 2014) or an item requiring negative concord (Nakatani, 2023), rather than a simple nominative-marked NP. For example, -*sika* ‘only’ attaches to a noun phrase as a suffix and requires the predicate to be negated, as in (16). Using sentences like (16), Nakatani (2023) found that the matrix verb *sinzi-nakat-ta* ‘did not believe’ was more difficult to process when the *sika*-marked subject was distant from the verb. That is, a subject-verb locality effect was observed. No comparable effect was found when the subject was a usual nominative subject or marked with -*dake* ‘only’, which is semantically similar to -*sika* but does not require a negative predicate.

(16) Tentyoo-sika [ueetoresu-ga zyoorenkyaku-o  nagut-ta-to] sinzi-nakat-ta
    manager-only [waitress-nom regular.customer-acc hit-past-that believe-neg-past
    node
    because
    ‘Because nobody but (the) manager believed that (the) waitress hit (the) regular customer,
  …’

Though CLT per se does not predict the contrast between *sika*-marked and ordinary subjects, it can be explained as an informational locality effect (Futrell, Gibson, & Levy, 2020; Hahn et al., 2022) under CCG-based parsing. The maximally incremental parse of (16) is the following (the internal structure of the complement clause is omitted):

(17) 
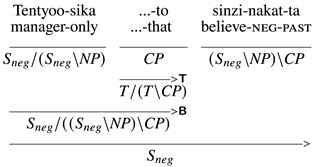

We assume that a *sika-*marked subject is inherently type-raised to select a negated verb phrase, *S*_*neg*_\*NP*. This means that the parser predicts a negated verb phrase when the *sika*-marked subject is encountered. This prediction would facilitate the processing of the negated verb downstream (Hale, 2001; Levy, 2008). However, this subject cannot be combined with the subsequent input until the complement clause is completed, as in a previous example (15). The subject expecting a negative verb phrase should then suffer from decay and/or interference while the complement clause is processed, and the precise information of the subject can be lost in some trials. If the *neg* feature on the subject is forgotten, the facilitatory effect at the verb would disappear. Since this forgetting is more likely to occur when the subject is held longer in memory, the loss of the facilitatory effect surfaces as a locality effect. This explanation is in line with the data reported by Nakatani (2023), where the locality effect seems to arise as a result of exceptionally fast reading time when the subject is *sika*-marked and adjacent to the verb, rather than a slowdown when the subject is *sika*-marked and distant from the verb.

In sum, CLT predicts the results of some previous controlled reading experiments regarding locality effects in Japanese, and is at least compatible with some other results. This contrasts with DLT, which does not predict the lack of locality effect in simple verb-final structures.

While DLT and CLT make divergent predictions with regard to the integration cost (locality effect), they make more similar predictions with regard to the storage cost. In DLT, storage cost is defined as the number of syntactic heads required to complete the current sentence. In most cases, this equals to the number of CCG constituents because each CCG constituent expresses the prediction for a constituent to combine with. Consider (15). When the subject *kisya-ga* ‘reporter-nom’ and the genitive *yotoo-no* ‘ruling.party-gen’ have been processed, two CCG constituents, *T*_1_/(*T*_1_\*NP*) and *NP*/*NP* are in storage. They express the expectation for a head verb and a head noun, respectively. Thus, in both DLT and CLT, the storage cost at this point is two. When the object *giin-o* ‘senator-acc’ is processed, CCG combines the argument cluster to a single constituent *T*_1_/((*T*_1_\*NP*)\*NP*), which awaits for the head verb. Again, at this point, the storage cost is the same in DLT and CLT: one. DLT and CLT storage costs diverge when multiple heads are predicted ahead that are dependent on the same, already seen head. In such cases, DLT storage cost but not CLT storage cost increases as more heads are predicted. Such cases are rare in Japanese syntax, as it is strictly head-final. Where they still diverge will be described in the Correlation Between Predictors subsection of the Corpus-Based Experiment section.

## CORPUS-BASED EXPERIMENT

In this section, we compare the contributions of DLT and CLT in predicting variance in reading times, using naturalistic reading data based on a Japanese corpus. Testing both theories with a head-final language like Japanese is important since their predictions diverge drastically in head-final languages, as described above. The code for the calculation of the predictors and for the statistical analysis reported below is available at https://osf.io/62utg/.

### Materials

We used the BCCWJ-SPR2 dataset (Asahara, 2022) to evaluate the prediction of CLT for naturalistic texts in Japanese and compare it with that of DLT. BCCWJ-SPR2 is a dataset of reading times collected online from native speakers of Japanese. The dataset uses texts of the Balanced Corpus of Contemporary Written Japanese (BCCWJ) (Maekawa et al., 2014). Reading times were collected using the non-cumulative, moving window self-paced reading method (Just et al., 1982). The texts were divided into *bunsetsu*, a group of morphemes that must be pronounced together (Hashimoto, 1934), and one bunsetsu was presented at a time. A bunsetsu is shown as a single orthographic word in the transcription used in this paper (i.e., bunsetsu boundaries are indicated by spaces). The participant pressed a key to proceed to the next word, and the time between one button press and the next was recorded as the reading time for that bunsetsu.

This study analyzes a subset of data from BCCWJ-SPR2 that uses the texts for which their Universal Dependencies (UD) annotations (de Marneffe et al., 2021; Nivre et al., 2020) are published (Asahara et al., 2018; Omura et al., 2021).^4^^,^^5^ By calculating both DLT and CLT costs from the same UD annotation, we ensure that the difference in the prediction of these theories is due to the theories, not due to any accidental differences in how the respective automated parsers for dependency and CCG resolve structural ambiguities. It also allows the same metric to be applied in DLT and CLT when measuring the distance of a string that intervenes in a non-local integration. We developed a simple pipeline Python script to calculate DLT and CLT costs from the UD annotation, which can be used in future studies to test the predictions of these theories for human behavioral or neural data associated with any linguistic resources with UD annotation. The materials in the subset all belong to the PB register of the corpus, which is a collection of texts taken from books of various genres. The materials consist of 83 *samples*, each of which is an excerpt from a book.

We randomly divided the 83 samples into 41 *exploratory* samples and 42 *confirmatory* samples of roughly the same text size. This is because there are multiple possible implementations of DLT and CLT, depending on how to measure the distance and what rules are assumed for CCG, as described below, and it is difficult to decide theoretically which implementations are the best. We first tested those multiple possible implementations against the exploratory samples, and the best-performing variant was submitted to the analysis with the confirmatory samples to ensure that the significant effects are not unique to the particular data. This way, we attempt a fair comparison of the core ideas of the two theories (Shain et al., 2022).

Before any analysis, data points that match one of the following conditions were excluded:

- The bunsetsu is the first or the last bunsetsu of the sentence
- The reading time is below 150 milliseconds (ms) or above 2,000 ms
- The participant got the true/false comprehension question that followed the sample wrong
- The participant’s overall comprehension accuracy is below 70% (the mean comprehension accuracy was 80.9%).

After these exclusion criteria were applied, if a participant provides less than 1,000 data points in the exploratory and confirmatory dataset, that participant was entirely excluded from that dataset. The amount of data after these exclusion processes is summarized in Table 1.

**Table 1.** The amount of data analyzed.

	**Exploratory**	**Confirmatory**
Data points	3,713,046	3,951,006
Participants	302	299
Samples	41	42
Sentences	4,636	4,509
Bunsetsu	30,699	34,158

### Predictors

In this subsection, we introduce the predictor variables used in the current analysis. They can be grouped by several criteria, and an overview is provided in Figure 2. First, the predictors can be divided into those of theoretical interest and control predictors. The former are integration and storage costs according to DLT and CLT, and are meant to model memory costs. The latter are included to control for variance in reading times that are due to known factors other than memory costs, such as word length and expectation. Second, cross-cutting this division is the distinction between dependency-based, CCG-based, and non-grammatical predictors. DLT and CLT costs are dependency-based and CCG-based, respectively, and most control predictors are non-grammatical. But we also have a grammar-based control predictor called *integration count*. Why we need this will be described below. Third, grammar-based predictors come with variations due to some details of implementation.

**Figure 2.**
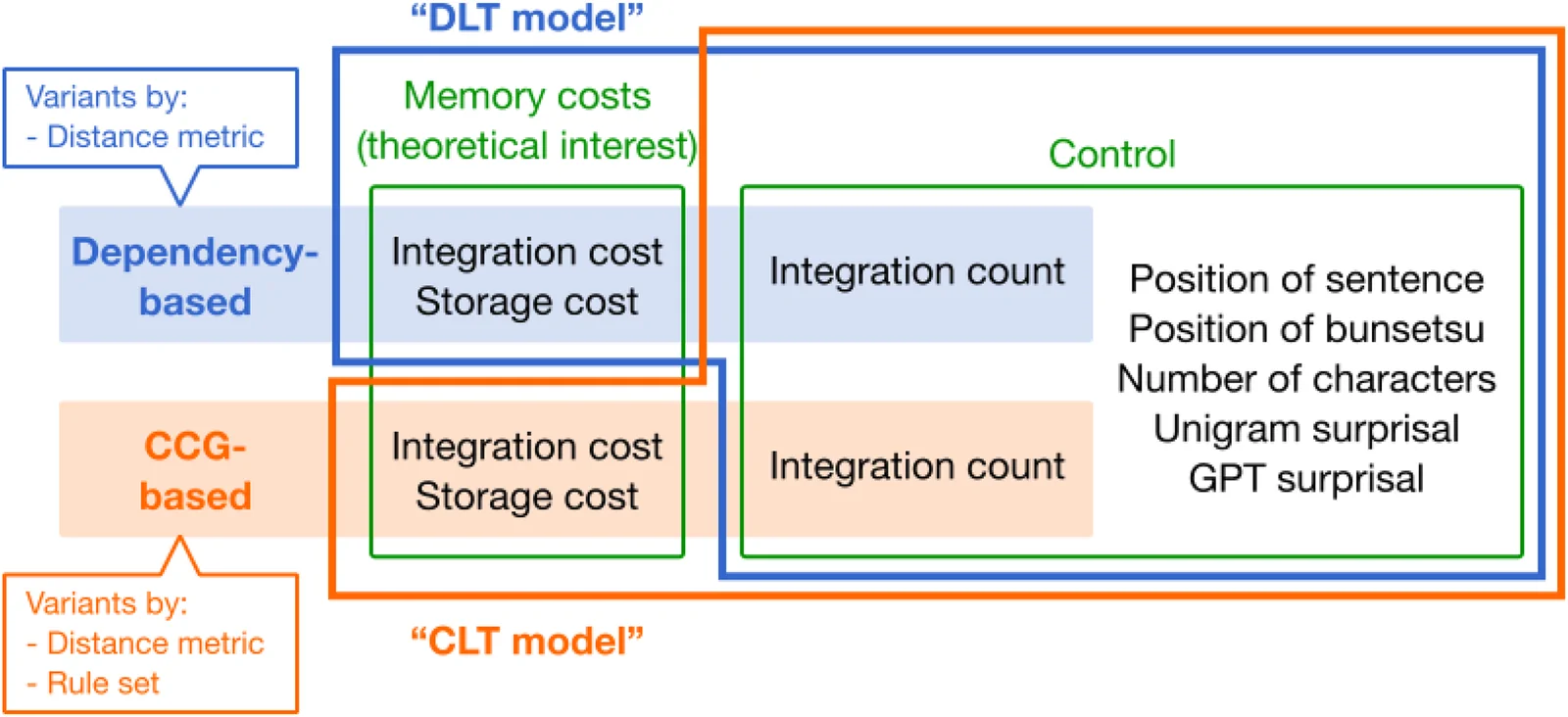
An overview of the predictors used in the statistical analysis.

#### Predictors of Memory Cost.

As described earlier, Both DLT and CLT define two metrics of processing difficulty (the labels in parentheses indicate shorthands used in formulas and figures):

- *Integration cost* (dlt/clt_int) models the locality effect. An integration cost is incurred when an integration of older and newer elements takes place. The size of the cost is determined by the distance between the elements. The distance is assumed to reflect the degree of the degradation of the older element, which depends on the amount of cognitive resources (time and/or attention) consumed to process the intervening element.
- *Storage cost* (dlt/clt_stor). This is incurred when the parser has to maintain multiple syntactic objects for future integration. In CLT this is the number of CCG constituents that are yet to be connected. In DLT, this is defined as the number of unseen heads required to complete the current input as a grammatical sentence. Since calculating it exactly is difficult as it involves judgment of grammaticality of hypothetical continuations, it is operationalized instead as the number of unseen heads that head or depend on head that are already seen.

To calculate these costs, we first need to identify the syntactic parses for the sentences in the dataset. For DLT costs, we simply use the UD annotation included in the original dataset. For CLT costs, we obtained the CCG parses by conversion from the UD annotation. The conversion process is detailed in the Appendix I. In short, the conversion was done by interpreting each dependency as a CCG application (>, <); the direction of the application was judged by the direction and the type of the dependency. The conversion process is highly generalized and is based on the conceptual relation between dependency grammar and CCG rather than the details of the Japanese grammar. The same method can be therefore applied to UD-annotated texts in any language in future studies. The normal form CCG trees thus obtained were then “rotated” recursively to obtain the most incremental tree possible. This incrementalization process has variants depending on the set of rules available.

We also need to specify some implementational details of the theories. Specifically, both CLT and DLT need a distance metric, i.e., a way to quantify the non-locality of an integration; CLT also needs a specific rule set (see Table 2 for the full structure of the choices). These implementational details should be distinguished from the core idea of CLT and DLT that CCG- or dependency-based distance predicts locality effect (Isono, 2024). Since we want to test the core claims, and the best implementation depends on the data we are dealing with (described below), we search for the best implementation in the exploratory stage and then test its performance in the confirmatory stage.

**Table 2.** Delta log likelihoods of the models considered in the exploratory analysis. The best-performing models (with the highest log likelihood) are highlighted with **bold**. NV: 1 distance unit per noun and verb; NVJR: 1 distance unit per noun, verb, adjective, and adverb; Char: 1 distance unit per character.

		**NV**	**NVJR**	**Char**
DLT		1308	1272	**1401**
CLT	without >**B**^2^ (B1)	1571	1490	**1608**
	with >**B**^2^ (B2)	1475	1434	1543

The distance metric reflects the assumption about the amount of computational resources consumed to process the intervening element. It is essentially an approximation, and one way of counting that worked for one language may not necessarily be appropriate in another. For example, even if counting nouns and verbs may have worked for English, it may not work for Japanese, given that the nature of nouns and verbs differ between these languages. For this reason, we consider several possible distance metrics. We try different metrics not only for CLT but also for DLT for a fair comparison, although the original DLT (Gibson, 2000) is committed to a specific metric. Specifically, we consider the following three distance metrics:

- The number of nouns (with UD part-of-speech tags NOUN and PROPN) and verbs (VERB) (this version will be indicated with the suffix _nv); this is close to the original metric used by DLT (Gibson, 2000)
- The number of nouns, verbs, adjectives (ADJ) and adverbs (ADV) (suffix _nvjr); this is close to the metric used by the original version of CLT (Isono, 2024)
- The number of characters (suffix _char)

The choice of CCG rule set similarly reflects the assumption about the level of incrementality, which may depend on factors such as the language, the difficulty of the secondary task, the population, etc. We consider the following rule sets:^6^

- >, <, >**T**, >**B** (suffix _B1)
- >, <, >**T**, >**B**, >**B**^2^ (suffix _B2)

The first set contains the most basic rules that are needed to parse dependents on the same head (e.g., the subject and the object of a clause-final verb) incrementally, for which there is much psycholinguistic evidence (Kamide et al., 2003; Kamide & Mitchell, 1999). The second set extends it with the >**B**^2^ rule, which is a more “powerful” variant of >**B** that allows two arguments (instead of one) to be inherited to the output category:

(18) 
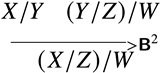

Figure 3 illustrates the calculation of the predictors with an example based on the actual corpus sentence.^7^ In DLT, the integration cost concentrates on the verb. The DLT integration cost for the subject-verb dependency (the blue arrow labeled as nsubj), for example, would be 2 if intervening nouns and verbs are counted (‘thing’ and ‘you’), or 9 if the letters of the intervening material are counted (from ‘TOP’ to ‘to’). The dependency also imposes storage cost of 1 (for one predicted verb) on the intervening elements. In CLT, the integration cost arises at the object and the oblique argument, as indicated by the orange arrows. The left orange arc integrating the subject and the object constituents imposes an integration cost of 1 if intervening nouns and verbs are counted (‘thing’) and 5 if characters are counted. The subject constituent also imposes a storage cost of 1 (for 1 constituent) until it is integrated with the object, i.e., on the elements covered by the left orange arc.

**Figure 3.**
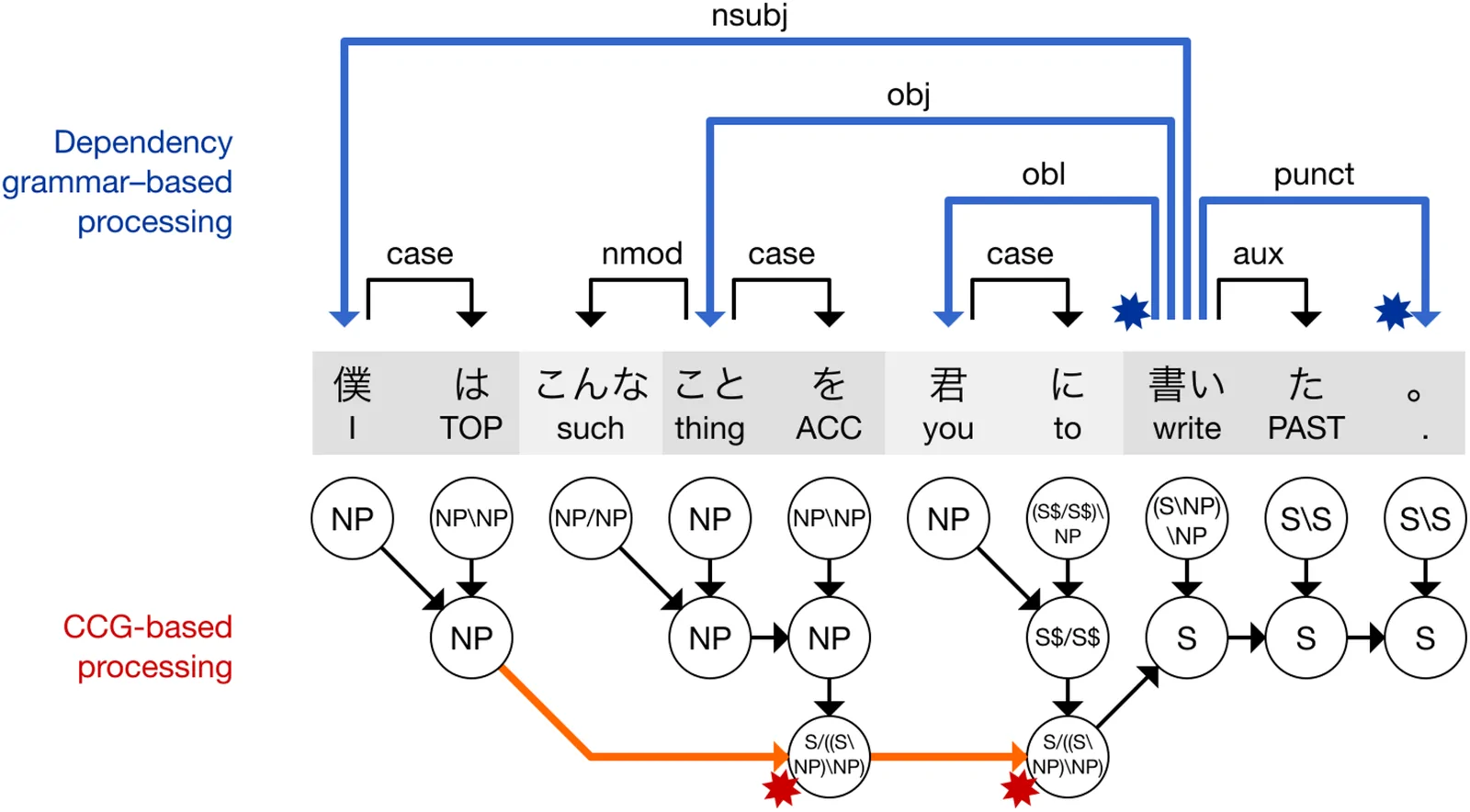
Dependency- and CCG-based processing of an SOV sentence in Japanese, taken from the BCCWJ-SPR2 dataset with modifications. Colored edges indicate non-local integrations. Octagram stars indicate where a non-zero integration cost is predicted. The regions covered by the colored arcs/edges are where a non-zero storage cost is predicted. The grey background for the text indicate the group of morphemes (bunsetsu) that were presented at a time.

#### Control Predictors I: Integration Count.

In addition to the predictors of theoretical interest described above, a number of control predictors are included in the regression models. The only grammar-based control predictor is *integration count*, defined below:

- *Integration count* (dlt/clt_cnt): the number of integrations (dependency completion in DLT; binary rule application in CLT) made at the region in question.

Integration count is included in the model for the following consideration. Integration *cost* is defined as the sum of the distance of the integrations made at the region (this definition originates in DLT (Gibson, 2000) and CLT follows it). By definition, then, it should correlate with the integration count of that region. We would like to distinguish these two factors not just because they are conceptually distinct, but also because they may have effects in the opposite direction, canceling each other out, as described below.

Dependency-based integration count is known to have a negative effect on reading times (Asahara, 2022; Asahara et al., 2019; Isono, 2024). The putative reason for the negative effect is that it is a proxy for the predictability of the word (Konieczny, 2000; Konieczny & Döring, 2003; Vasishth & Lewis, 2006). Integration count of *n* at word *w* means that there are *n* words that have a dependency relation with *w* (either the head or a dependent of *w*). These words may be informative in predicting *w*. For example, the presence of *eat* in context makes an edible object noun more likely to appear. Since predictable words are processed more quickly, it is reasonable to expect that words with larger integration counts are read more quickly. Isono (2024) showed that controlling for this confound is important in evaluating DLT: whlie Dotlačil (2021) previously observed a negative effect of DLT integration *cost* (distance) in the Natural Stories corpus, Isono observed that when DLT integration *count* was also included in the model, the distance had no significant effect while the count had a significant negative effect.^8^

For the same reason, we speculated that CCG-based integration count has a negative effect on reading times and controlled for it in the statistical modeling. If the human parser compresses the syntactic information of the context into CCG constituents, as postulated by CLT, the number of CCG integrations that take place at a region reflects the amount of such information that is relevant for the current region. Unlike dependency-based integration count, there has not been clear evidence for the effect of CCG-based integration count. Several studies have investigated CCG *node count*, (Kajikawa et al., 2024; Ozaki et al., 2024; Stanojević et al., 2023), which differ from integration count in counting unary rules such as type-raising. We exclude unary rules from integration count since only binary rules, not unary rules, can increase integration cost (because unary rules are not integration of two elements) and can be correlated with predictability.

After the costs and counts were calculated for each token in the dependency parse, they were aggregated at the bunsetsu level by summing (integration cost/count) or taking the value of the last token (storage cost). For example, in Figure 3, the CCG integration costs for ‘thing’ (5, character-based) and ‘acc’ (0) are summed to give the CCG integration cost for the bunsetsu ‘thing-acc (5 + 0 = 5). The CCG integration count for the same bunsetsu is 3: integration of ‘such’ and ‘thing’, ‘such thing’ and ‘acc’, and finally ‘I-top’ and ‘such thing-acc’. The CCG storage cost for the same bunsetsu is 1, since when the bunsetsu is completed by acc, there is just one CCG constituent.

Like CLT integration/storage costs, CCG-integration count varies depending on the rule set. When evaluating CLT integration/storage costs under one rule set, CCG-based integration count from the same rule set is used as control.

#### Control Predictors II: Non-grammatical Predictors.

We also included several other non-grammatical control predictors in the regression model that are available for the current dataset and often included in corpus analysis (Demberg & Keller, 2008; Demberg et al., 2013; Dotlačil, 2021; Isono, 2024; Shain et al., 2016). They are listed below.

- *The position of the sentence* in the sample (SPR_sentence_ID). This is expected to have a negative effect on reading times since participants of reading experiments usually speed up towards the end of a coherent text and also through the course of a reading experiment.
- *The position of the bunsetsu* in the sentence (SPR_bunsetsu_ID). This is also expected to have a negative effect since participants often speed up towards the end of a sentence.
- *The number of characters* in the bunsetsu (nchar). This should have a positive effect since longer texts should take longer to read.

We also included two kinds of *surprisal* values listed below. According to the surprisal theory (Hale, 2001; Levy, 2008), a word is more difficult to process when it is less expected. This difficulty is quantified as the surprisal value −log P(*w*|*C*), where P(*w*|*C*) is the probability of the word *w* given the context *C*. The surprisal values are expected to have a positive effect on reading times.

- *The sum of unigram surprisal* of the morphemes comprising the bunsetsu (uni_surp). In analysis of corpus-based reading time data, it is commonplace to include the frequency of the word to the statistical model. Doing this for a Japanese corpus is not straightforward because a bunsetsu often consists of many morphemes, and observing the exact bunsetsu can be quite rare even if it consists of frequent morphemes. This study instead considers the morpheme-level rarity of the text by using the sum of unigram (i.e., decontextualized) surprisal of the morphemes comprising the bunsetsu. The probability of each morpheme is taken from the unigram frequency table of the NINJAL Web Japanese Corpus (Asahara et al., 2014)^9^.
- *Surprisal values from GPT-2* (gpt_surp), a Transformer-based large language model (Radford et al., 2019). We used the smallest model of the Japanese GPT-2 (japanese-gpt2-xsmall) published by rinna Co., Ltd. (Sawada et al., 2024).^10^ Surprisal values from the smallest (“extra-small”) model showed a better fit to the current corpus data compared to those from the small- and medium-sized models. This is consistent with previous reports that smaller GPT models are better at capturing reading times (Oh & Schuler, 2023; Shain et al., 2024). The model does not directly give bunsetsu-by-bunsetsu surprisal values because it relies on tokenization defined by the sentencepiece tokenizer (Kudo & Richardson, 2018). To address the mismatch, the token-based surprisal values were evenly distributed to characters comprising the token, and then this by-character surprisal values were summed up to obtain the by-bunsetsu surprisal values.

This completes the list of predictors considered in the regression analysis. See Figure 2 for an overview.

#### Correlation Between Predictors.

Figure 4 shows the correlations between the variants of DLT and CLT predictors. We can see that the variants of the same predictor are similar to each other, as their correlations are generally very high (*r* > 0.9).

**Figure 4.**
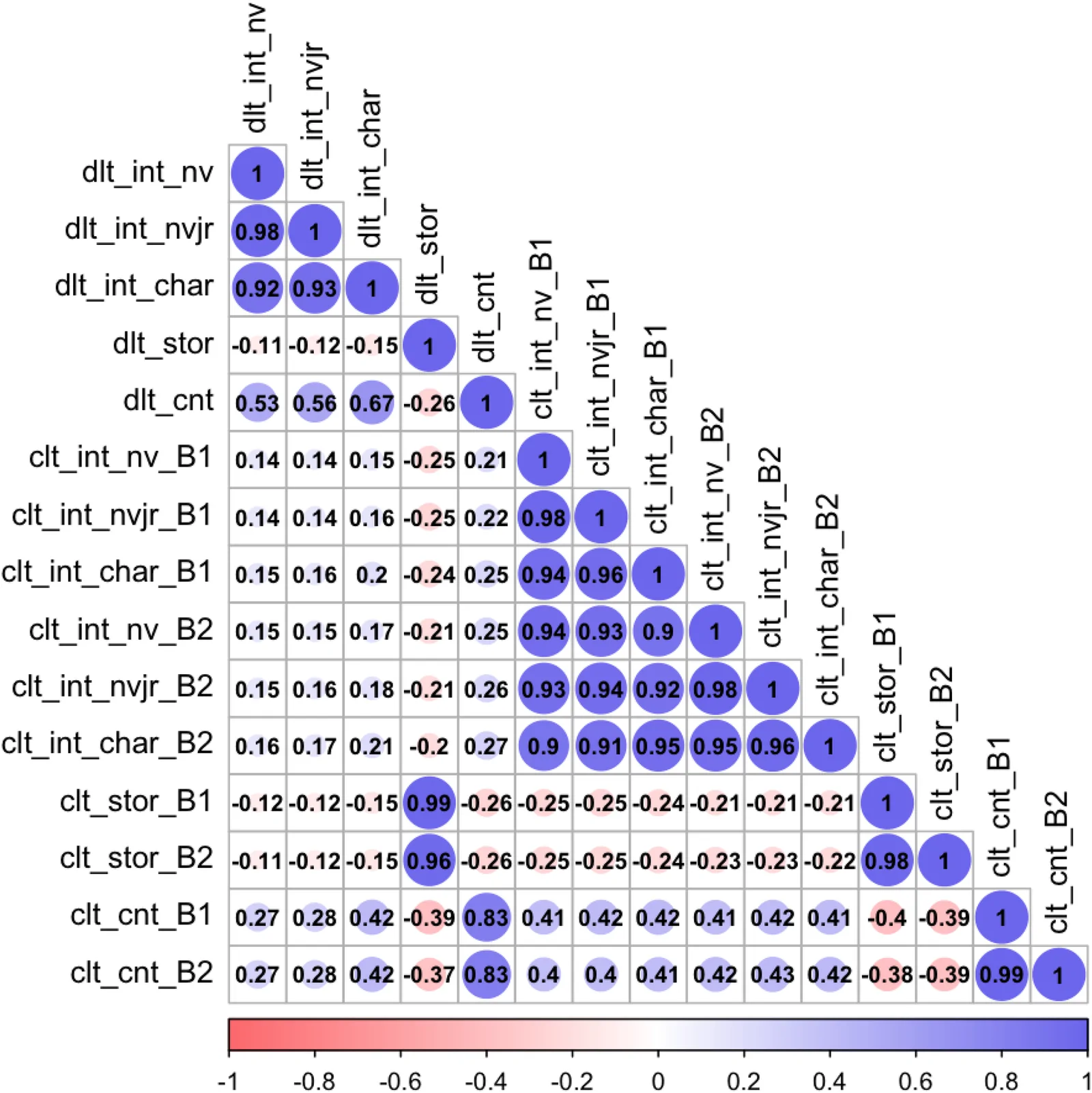
Correlations between variants of DLT and CLT predictors.

Figure 5 shows the correlations between the predictors used in the confirmatory analysis, i.e., the best-performing DLT and CLT predictors and the control predictors. Note the low correlation between DLT and CLT integration costs (*r* = 0.2). This confirms our earlier theoretical discussion that the two theories make divergent predictions regarding integration costs. In contrast, DLT and CLT storage costs are highly correlated (*r* = 0.99).

**Figure 5.**
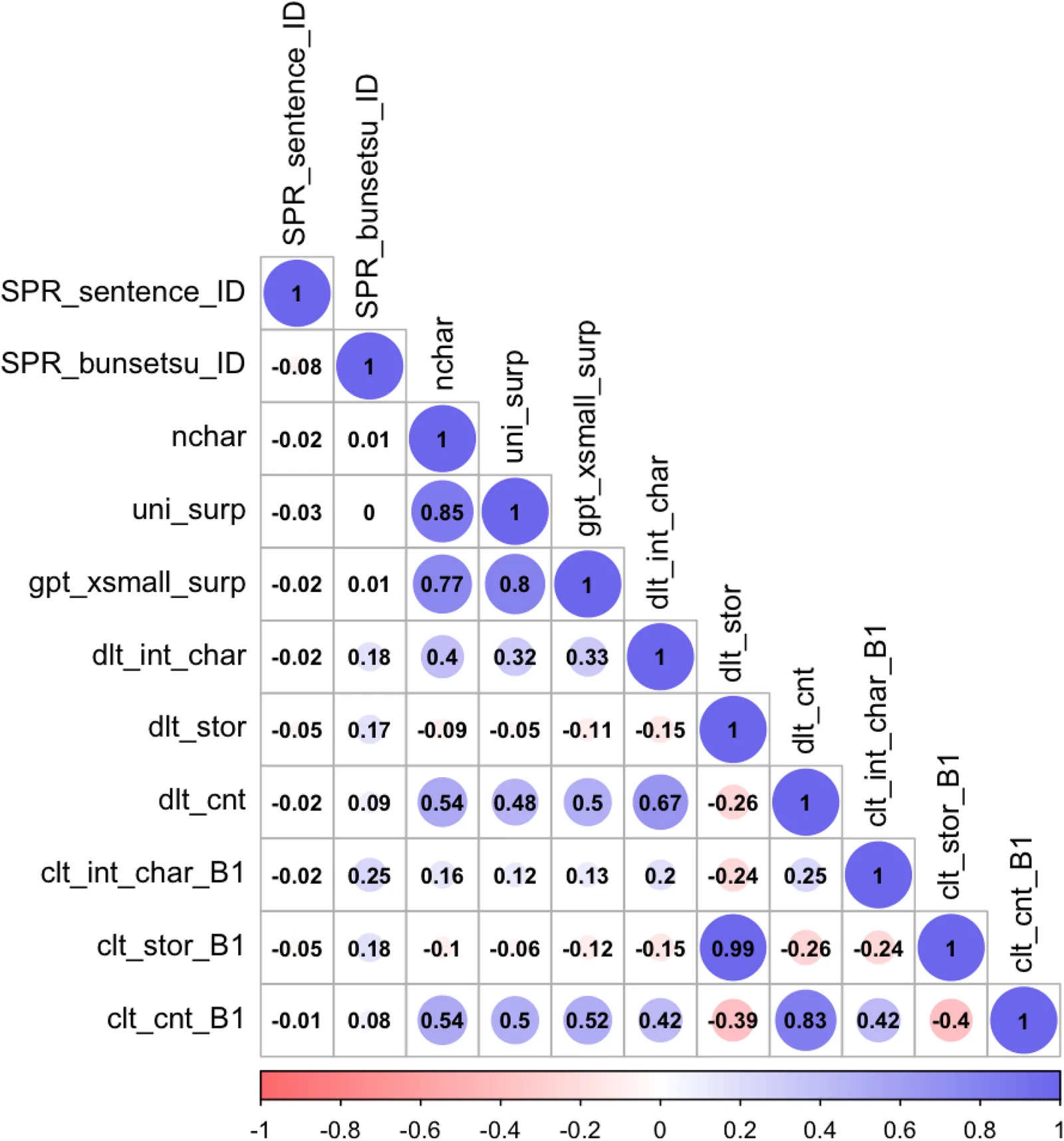
Correlations between predictors used in the best-performing model.

Before proceeding, we touch on the relationship between CLT and DLT storage costs. In the current dataset, they are highly correlated, and they in fact match in most regions. This is theoretically expected, as discussed earlier. Incremental CCG parsing group words into constituents if they are dependent on the same predicted head, while DLT counts the number of predicted heads according to the dependency grammar. In a head-final structure, they essentially count the same thing. They diverge, however, in a number of situations. First, when the head is right-dislocated, dependents can follow. This increases DLT storage cost, since each predicted dependent counts, but not CLT storage cost, since post-head dependents are expressed as part of the head’s category (e.g., *Y* and *Z* in the head category (*X*/*Y*)/*Z*). Second, in the Universal Dependencies (UD) annotation which we use in the corpus analysis, suffixes are analyzed as dependent on the stem. Since Japanese morphology is suffix-heavy, this creates many cases where multiple dependents follow a single head. This divergence has limited impact on the corpus analysis below since we generally ignore bunsetsu-internal dependencies. The exception is when the suffixes are split into multiple bunsetsus, which happens when a quotation mark intervenes (as in *“kisya-da” -to itta* ‘said that «(that) is a reporter»’, where the complementizer suffix *-to* is analyzed as a single bunsetsu). Third, crossed dependencies in the original UD annotation result in disagreement between DLT and CLT since they are fixed in the process of UD-to-CLT conversion for technical reasons (see Appendix I for details). Fourth, the original UD annotation contains other miscellaneous cases where more than one dependents are attached to a preceding head, leading to an increase in DLT storage cost but not CLT storage cost. This includes parsing errors and corner cases where the correct parse is not obvious. This is concerning, but fixing all of such cases in a large-scale dataset is not realistic and can lead to arbitrariness. Thus, while CLT and DLT storage costs are conceptually different and can make different predictions in non-head-final environments, the difference is minor in the current dataset. We do, however, argue that CLT storage cost is conceptually more natural; see the Discussion section on this point.

### Statistical Analysis

Our goal is to test whether the predictors of memory cost provided by CLT and DLT explain a variance in reading times that is not explained by other known predictors. Toward this goal, we initially conducted a simple linear mixed-effects modeling, following some previous work analyzing naturalistic reading time datasets (Demberg & Keller, 2008; Dotlačil, 2021; Isono, 2024). However, we faced a number of problems, and switched to a different approach. Most importantly, we could not fully appreciate variations between participants, and that motivates the final analysis, described below. The initial analysis is reported in Appendix II for transparency, and will also be mentioned below since some of the problems motivated the final analysis.

The final analysis consisted of two stages: exploration and confirmation. This is because both CLT and DLT come with variants concerning implementation details, as described earlier. The best performing variants identified in the exploration stage were used the confirmation stage. Non-overlapping subsets of the original dataset were used in these stages.

In each of these stages, the following analysis was conducted (Figure 6). First, data points from each participant was separately modeled by eight linear models: the baseline model containing just the control predictors; the DLT models containing DLT integration cost, storage cost, or both; the CLT models containing CLT integration cost, storage cost, or both; and the DLT + CLT model containing all the four costs (see Table 3). The models additionally contain predictor values from the previous region to model the spillover effect (Mitchell, 1984).^11^ All the predictors were *z*-transformed for the stability of model fitting. The reading times were log-transformed. These models were evaluated by 10-fold cross validation as follows. For each participant, the data was randomly split to 10 folds of equal size. In each of the 10 iterations, one of these folds was held out and the models were fit to the remaining nine folds. The log likelihoods (*LL*s) assigned by the models to the held-out data indicate their predictive fit. Accordingly, the increase in *LL* (Δ*LL*) when a predictor or a set of predictors is added to the model indicate the predictive power of the predictor(s) (Wilcox et al., 2023). In the exploratory stage, we looked at Δ*LL*s of the CLT models and DLT models compared against the baseline model to identify the best-performing variants. In the confirmatory stage, we made several comparisons addressing the theoretical questions. For each comparison, we conducted a permutation test (Wilcox et al., 2023): we took the distribution of the by-bunsetsu Δ*LL*s averaged over participants, and assessed whether the mean of Δ*LL*s is significantly above zero (i.e., one model performs better than the other), by generating a distribution of hypothetical mean Δ*LL*s by randomly flipping the sign of Δ*LL*s (equivalent to permuting the label for the model assigning the *LL* value for each bunsetsu) and locating the observed mean in this distribution.

**Figure 6.**
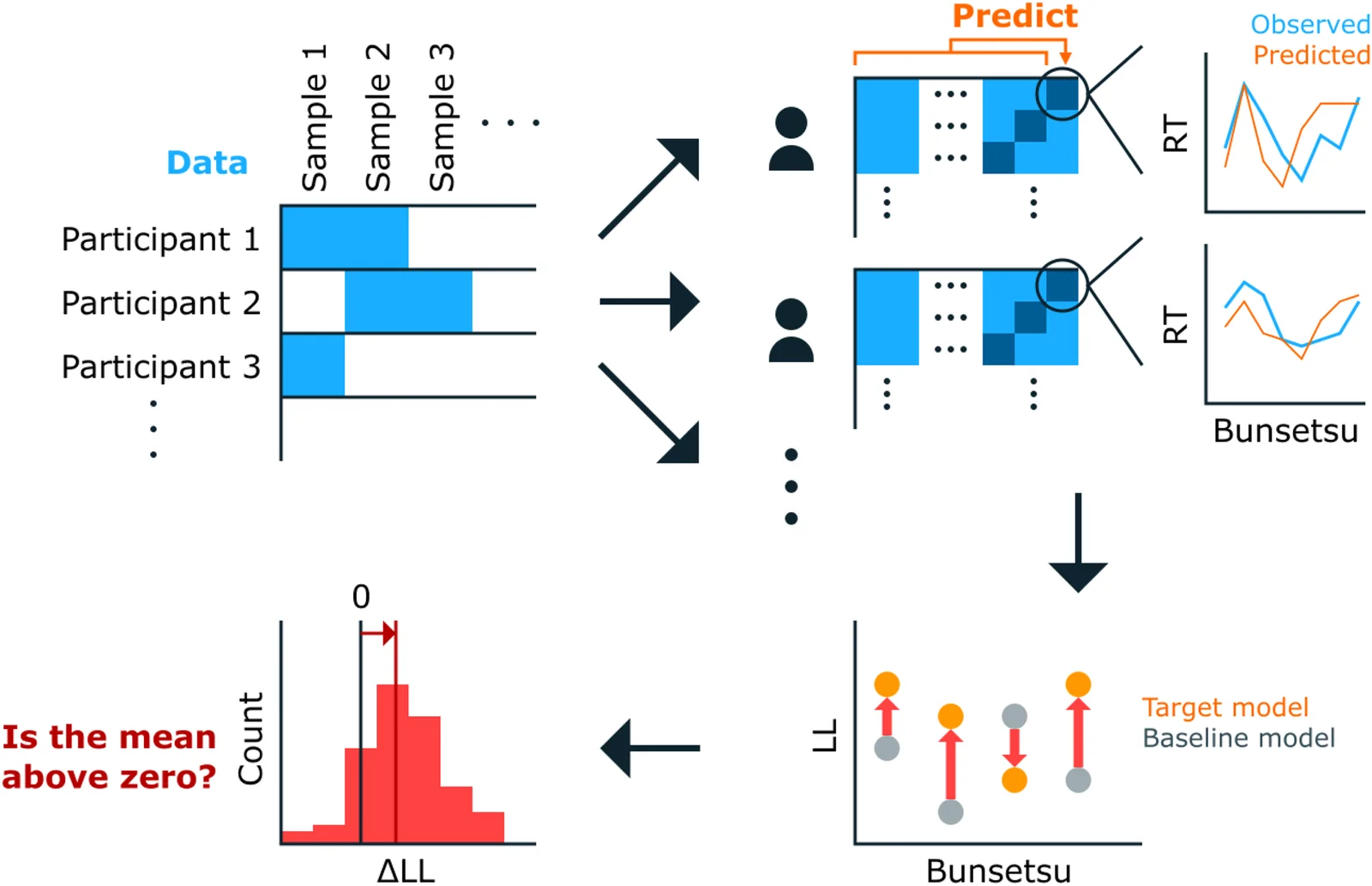
An overview of the analysis.

**Table 3.** Results of the confirmatory analysis. The checkmarks indicate the predictors included in the respective model, where “ctrl” stands for control predictors, “int” for integration cost, and “stor” for storage cost. The “comparisons” columns show the Δ*LL*s, which indicate the improvement of the predictive power of the model on the left compared to the model on the top. The stars indicate the result of the permutation test. Only pairs of models of theoretical interest are compared.

**Model name**	**Predictors included**					**Comparisons (Δ*LL*)**	
	**Ctrl**	**DLT_int_**	**DLT_stor_**	**CLT_int_**	**CLT_stor_**	**vs. Base**	**Others**
DLT_int_	✓	✓				−694	
DLT_stor_	✓		✓			2164***	
DLT	✓	✓	✓			1561***	
CLT_int_	✓			✓		138*	(vs. DLT_int_: 832***)
CLT_stor_	✓				✓	2219***	(vs. DLT_stor_: 55*)
CLT	✓			✓	✓	2293***	(vs. DLT: 732***)
DLT + CLT	✓	✓	✓	✓	✓	1366***	

*Note*. Significance code: *** for *p* < 0.001, * for *p* < 0.05.

This approach is motivated by a number of considerations. First, it allows the data from each participant to be modeled separately to fully appreciate between-participant variations. Human readers can vary in how they respond to various linguistic features (King & Just, 1991; Nicenboim et al., 2016). A common approach to take such variations into account is to fit a linear mixed-effects model (Baayen et al., 2008) to the entire dataset, with random effects addressing between-participant variations. This approach is standard in controlled experiments and is in fact taken in some previous studies analyzing naturalistic reading time data (Demberg & Keller, 2008; Dotlačil, 2021; Isono, 2024). We also took this approach at first, but the full model failed to converge. By modeling each participant separately, we avoid convergence issues while acknowledging that participants may respond differently to the factors considered here. Second, we ask whether the predictor of interest improves predictive fit to held-out data, rather than whether its overall effect for the entire dataset is significantly away from zero. Evaluating the predictive fit avoids spurious effects due to overfitting. Note that when by-participant modeling and evaluation based on predictive fit are combined, a predictor can be judged to be a significant contributor even when it has effects in opposing directions (slowdown or speedup) depending on the participant, as long as the effect is consistent within each participant. We return to this point in the subsection named “Additional analysis I.”

### Results

The result of the exploratory analysis is summarized in Table 2. Character-based distance was the best for both DLT and CLT, and the >**B**^2^ rule was not beneficial for CLT.

The result of the confirmatory analysis is summarized in Table 3. DLT integration cost worsened the model fit compared to the baseline model. This means that DLT integration cost was a noise for prediction. In contrast, DLT storage cost, CLT integration cost, and CLT storage cost showed significant improvements in the fit. When integration and storage costs were evaluated together, both DLT and CLT models showed significant improvements from the baseline model. When CLT and DLT models were compared, CLT was significantly better than DLT. The DLT + CLT model fitted worse than both the DLT and CLT models. This is probably because of the high correlation between DLT and CLT storage costs: multicollinearity makes the coefficient estimates for these variables less reliable, and can result in a lower predictive performance of the model. Overall, these results favor CLT over DLT.

We also plotted the distribution of Δ*LL* by aggregating datapoint-wise Δ*LL* at the bunsetsu level and the participant level (Figures 7 and 8) to see how general the improvement in predictive fit is. We see right skews in the participant-based plot, which suggest that the current predictors improve predictive fit for many participants. The skew is not visually evident in the by-bunsetsu plot. Note, however, the significant results of the permutation tests indicate that the current results are unlikely to be due to random outliers.

**Figure 7.**
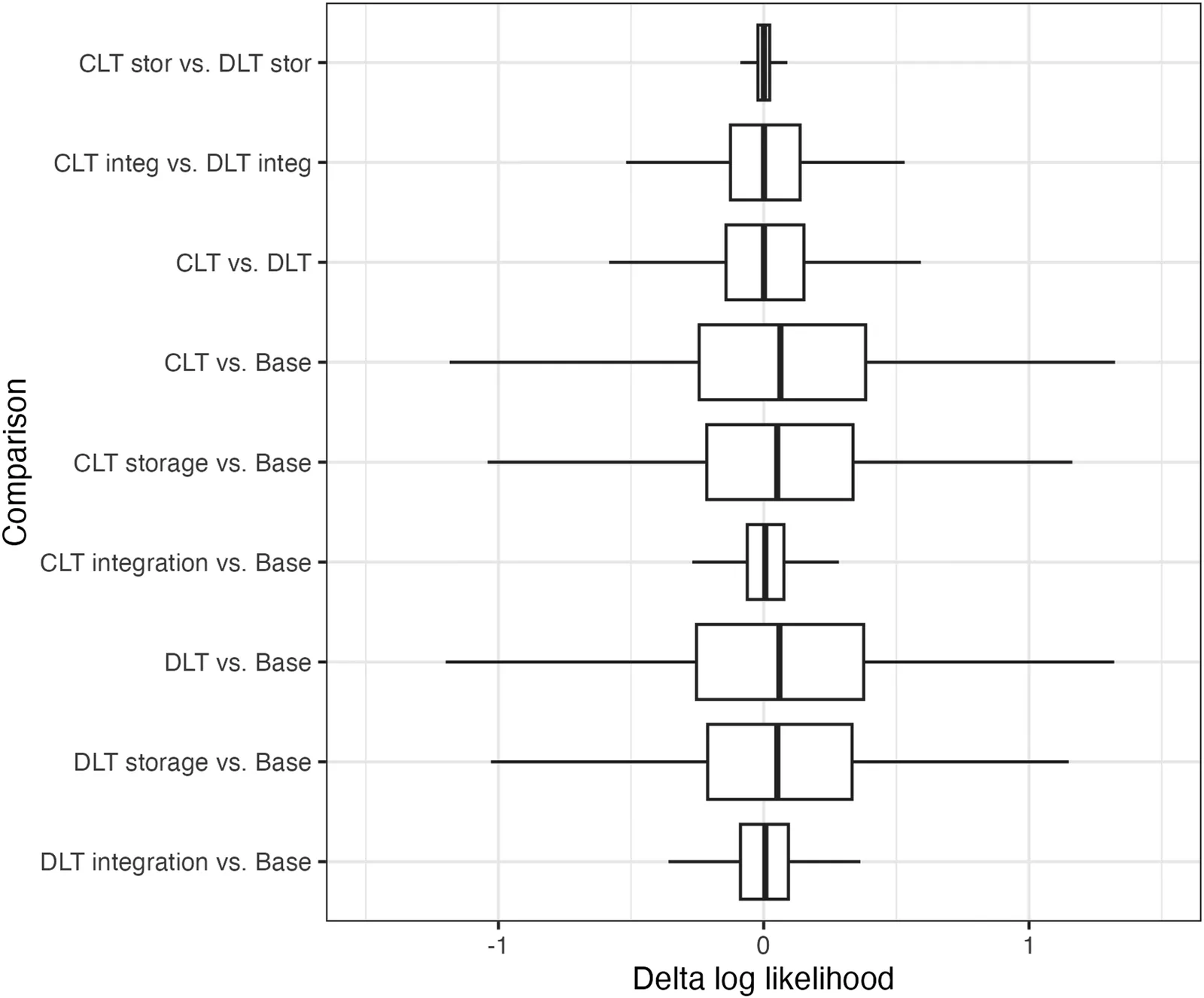
Boxplots showing the distribution of by-bunsetsu Δ*LL* across model comparisons. Outliers are not shown.

**Figure 8.**
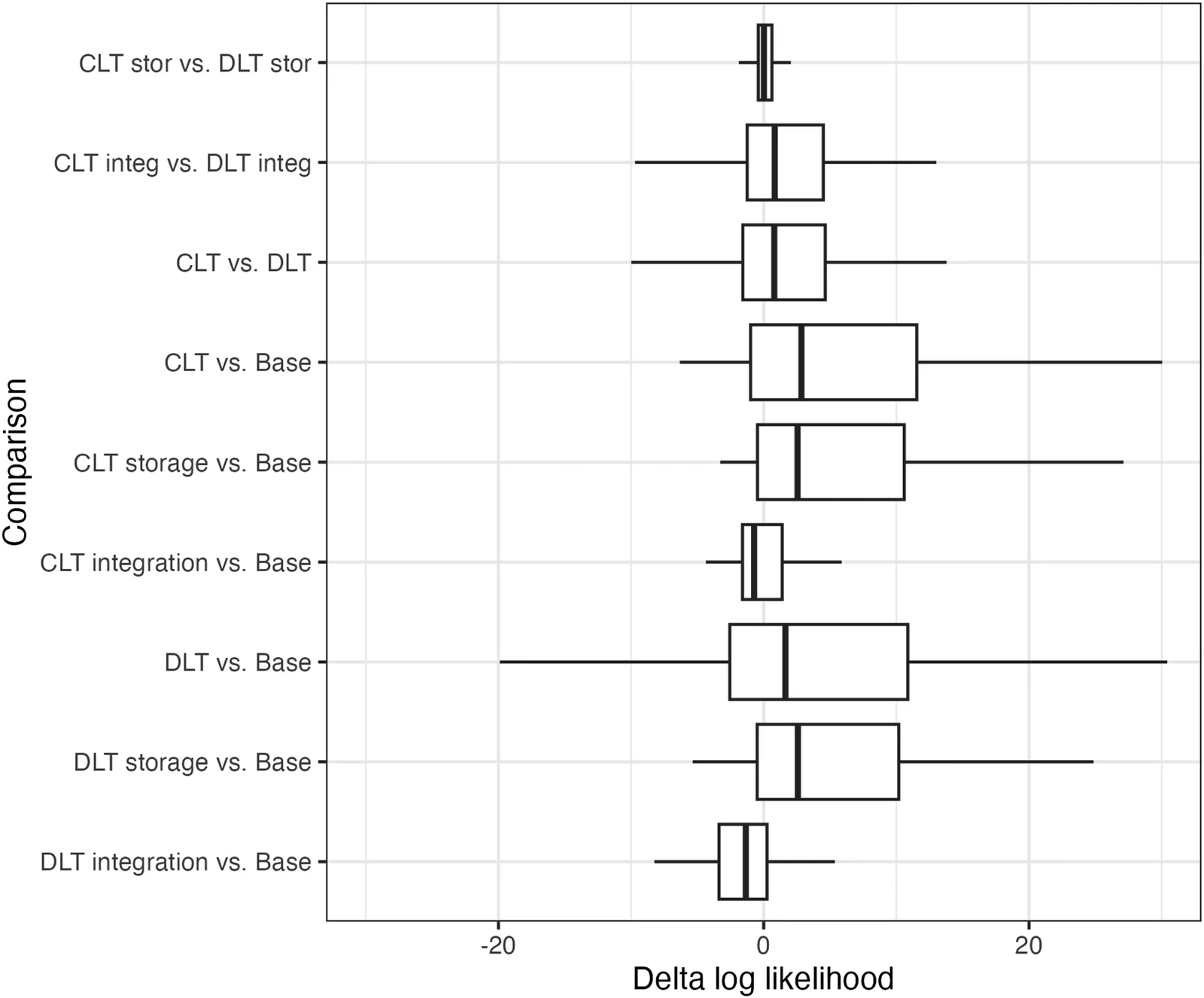
Boxplots showing the distribution of by-participant Δ*LL* across model comparisons. Outliers are not shown.

#### Additional Analysis I: The Direction of the Effects.

Tests based on Δ*LL* focus on improvements in predictive fit, and are agnostic about the direction of the effects. We therefore looked at the distribution of the estimated coefficients for the predictors of theoretical interest. The coefficients from 10 models from each participant (due to 10-fold cross validation) are averaged and plotted in Figure 9.

**Figure 9.**
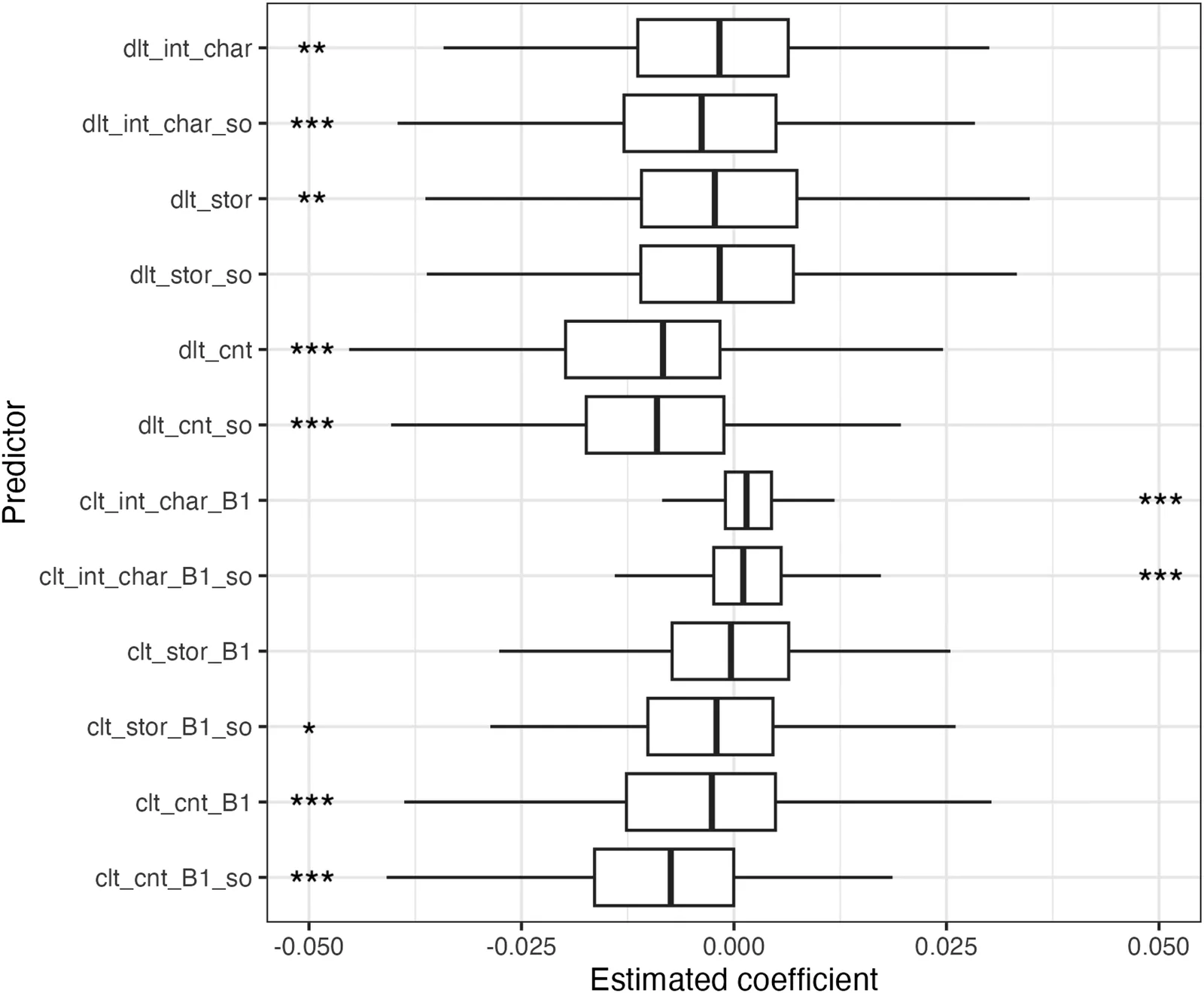
Boxplots showing the distribution of by-participant estimated coefficients for each predictor. Outliers are not shown. Stars indicate the results of permutation tests: ***p < 0.001, **p < 0.01, *p < 0.05.

DLT and CLT integration counts show relatively clear negative effects. This is consistent with the view that integration count is confounded with predictability, which is the reason why we included them in the regression models. It is also consistent with previous observations (Asahara, 2022; Asahara et al., 2019; Isono, 2024).

The effect of CLT integration cost is significantly positive, which is consistent with the view that it indexes the degree of degradation of CCG constituents and retrieving degraded constituents take time. The effect of DLT integration cost is significantly negative. A negative effect of DLT integration cost is also reported by Dotlačil (2021), but is inconsistent with the view that DLT integration cost is related to retrieval difficulty. Also, recall that DLT integration cost was not predictive according to the log-likelihood based test in the first place. A possible interpretation of these results is that DLT integration cost is confounded with some unidentified factor that has a negative effect on reading time, but the alignment is not close enough to give DLT integration cost a strong predictive power.

The results with DLT and CLT storage costs are interesting since, although they showed a larger predictive power than integration costs, the direction of the effects is less clear. If anything, the trend is negative. The original motivation for the DLT storage cost (which CLT adopts) predicts a positive effect since storage of items in memory should use cognitive resources and thus slow down processing (Gibson, 2000). The robust predictive power suggests that the effect of storage cost is consistent within each participant—though the direction of the effect is not necessarily consistent across participants. This is reminiscent of the observation by Nicenboim et al. (2016) that readers with relatively low memory capacity can speed up in memory-demanding structures. While their explanation for the speedup is based on retrieval failure, it is possible to think of a mechanism in which high storage cost leads to speedup (Isono & Kajikawa, 2026). A high storage cost means that there is much linguistic information that has to be carried on to integrate with future input. Slowdown is necessary to process the intervening material correctly with the remaining resource. But an alternative strategy that a reader can take is to rush to get out of the demanding structure and avoid the risk of losing the stored information. This can sacrifice the understanding of the intervening element, but some readers may find it good enough (Ferreira et al., 2002). Given the possibility of such between-participant variability, we believe that the weakly negative direction of the DLT/CLT storage costs do not immediately undermine the hypothesis. Admittedly, there remains a possibility that what we see as the significant predictive power of DLT/CLT storage cost reflects something very different from storage cost. One possible way to be more confident in the cost-based interpretation would be to see the correlation between the direction of the effect and the cognitive capacity of each participant. If lower capacity readers tend to speed up and higher capacity readers tend to slow down, it would reinforce the cost-based interpretation. But we leave it for future study since the current dataset does not contain information about working memory capacity of participants.

#### Additional Analysis II: What DLT and CLT are Good At.

We further investigated what drove the observed effects of DLT and CLT, as one reviewer asked. One possibility is that they captured processing difficulty of particular constructions. To test this possibility, we fitted new linear models to predict the Δ*LL* of each bunsetsu from the part-of-speech (POS) tags of its constituent tokens, taken from the UD annotation. Δ*LLs* of DLT storage cost, CLT integration cost, and CLT storage cost from the baseline were analyzed. Only POS tags that appear in 100 or more bunsetsus in the confirmatory dataset were considered. Since each bunsetsu contains to multiple tokens in the UD annotation, each bunsetsu was associated with a list of boolean values (0 or 1) indicating whether it contains a token with the POS tag. We did the same analysis with dependency type tags.

Table 4 summarizes the result of this analysis.^12^ The result shows that CLT integration cost is good at predicting reading times of bunsetsus with AUX(iliary) tokens. AUX covers a variety of functional suffixes that attach to a predicate, such as the copula *da*, the past tense marker *ta*, and the passive marker *rareru*. DLT and CLT storage costs are found to be good at PRON(ouns).

**Table 4.** The summary of the linear models predicting Δ*LL* for each bunsetsu by its part-of-speech and dependency type tags. Asterisks indicate significant effects of the dependency type.

	**DLT_stor_**	**CLT_int_**	**CLT_stor_**		**DLT_stor_**	**CLT_int_**	**CLT_stor_**
ADJ				acl			
ADP		(*)		advcl	*		
ADV		(*)		advmod		(*)	
AUX		*		aux		*	
NOUN				case		(*)	
PRON	**		**	mark			
PROPN				nmod			
PUNCT	(**)	(***)	(*)	nsubj	*		
SCONJ				obj	**		
VERB				obl	*		
				punct	(**)	(**)	(*)

*Note*. Significance codes: *** *p* < 0.001, ** *p* < 0.01, * *p* < 0.05, blank for nonsignificance. Parentheses indicate a negative coefficient (i.e., worse fit).

Then the next question is whether the predictive power of CLT are limited to these specific POS tags. This question is addressed by whether the addition of the POS tag information to the regression model eliminates the effect of the CLT costs. If the POS tag flag (0 or 1) eliminates the original effect, it means that the CLT cost in question only contributed to the categorical distinction between bunsetsus with and without that POS tag. If the interaction of the POS tag flag and the CLT cost in question eliminates the original main effect, it means that the CLT cost in question accounts for the variance within that POS tag, but not in others.

When the AUX flag was included as the baseline, CLT integration cost remained marginally significant (*p* = 0.060). The interaction of the AUX flag and CLT integration cost turned out to be merely a noise, as its addition worsens the baseline model. This result suggests that the predictive power of CLT integration cost may be partially due to its ability to categorically distinguish predicates and non-predicates, but not limited to it.

CLT storage cost remained clearly significant (*p* < 0.001) even after the inclusion of the PRON flag. The interaction of the PRON flag and CLT storage cost again turned out to be a noise. That is, we do not find evidence that the predictive power of CLT storage cost is limited to constructions involving particular POS tags.

## DISCUSSION

In this study, we investigated whether memory effects during the reading of naturalistic texts in Japanese can be predicted by two contrasting syntax-based theories: DLT, based on dependency grammar, and CLT, based on Combinatory Categorial Grammar (CCG). These two theories share the idea that complicated syntactic structures can tax working memory, but have different assumptions regarding what structures are taxing. DLT assumes that long dependencies (e.g., subject-verb dependencies) are difficult. CLT assumes that processing difficulty arises from failure of information compression based on logical inference about sentence structure. Both theories posit that two mental mechanisms incur processing cost: integration of new input to the past context, and storage (or maintenance) of the past context. Previous studies show that DLT integration cost has difficulty predicting reading times based on English corpora (Demberg & Keller, 2008; Dotlačil, 2021), while CLT integration cost can (Isono, 2024). Less has been known about the corpus-level effect of storage cost on reading times (cf. Isono, 2024). By using a head-final language, Japanese, we put these theories to a further test. Testing DLT and CLT in a head-final language is important because they make contrastive predictions as to where memory-based processing difficulties arise.

In line with previous results, we found that CLT integration cost was predictive of reading times, but failed to find reliable evidence for DLT integration cost. We also found that both CLT and DLT storage costs were predictive of reading times, and CLT storage cost was slightly better than DLT storage cost. Overall, these results favor CLT over DLT. We discuss some implications of the current results below.

### Memory-Constrained CCG as a Candidate for the Universal Processing System

The empirical success of CLT in the previous and current studies suggests that CLT captures some cross-linguistic, potentially universal aspect of real-time sentence comprehension. It is therefore worth reiterating the core ideas of CLT: (i) sentence comprehension is a process of logical inference which puts the input together while simultaneously predicting the future input; (ii) this process is directly guided by the grammar (CCG) which is a kind of logic; and (iii) this process is constrained by the capacity of working memory, which stores each step in the logical inference as a chunk.

Notably, some characteristics of the processing of head-final structures noted in the psycholinguistic literature fall out from these general assumptions. It has been observed that pre-head processing plays a key role in head-final structures (Kamide et al., 2003; Kamide & Mitchell, 1999; Nakatani, 2023; cf. Pritchett, 1992). Pre-head processing is predicted by CCG since the grammar itself is equipped with combinatory rules which allow arguments and adjuncts to be combined into single constituents before the head is seen. Furthermore, CLT correctly predicts that memory-based processing cost of a head-final structure is not concentrated on the head, as observed in previous experiments (Levy & Keller, 2013; Nakatani & Gibson, 2010; cf. Safavi et al., 2016). This CCG-based, universalist account contrasts with an alternative account of the lack of processing difficulty at clause-final verbs, according to which speakers of verb-final languages have a special strategy or capacity that allows them to process long dependencies without much difficulty (Levy & Keller, 2013; Nakatani & Gibson, 2010).

### Is Dependency Locality Unimportant?

While the failure of DLT integration cost in the current study is in line with previous reports based on English corpora (Demberg & Keller, 2008; Dotlačil, 2021; Isono, 2024) and Japanese controlled experiments (Isono & Hirose, 2023; Nakatani & Gibson, 2010), it may appear puzzling or dubious given the straightforwardness of the idea that longer dependencies are more difficult to process. It should be emphasized that the current result does not deny that dependencies are relevant for processing. In some trivial sense, it is of course essential for comprehension to establish the relation between, for example, the head noun of a subject and the verb. It is probably the case that the verb triggers retrieval of the subject, as usually assumed. But that does not necessarily result in a locality effect, as predicted by DLT. If the subject is activated elsewhere before the verb is seen, for example, at the object (in an SOV language), the subject-verb locality effect would not arise at the verb. In other words, though dependency probably is important in comprehension, the parser can integrate words more eagerly than suggested by dependency-based processing. CLT pinpoints elements that are still difficult for the parser to integrate because of the complexity of the logical inference required.

Nor is the current result contradictory to the suggestion that grammars and productions of natural languages tend to minimize dependency length (Futrell, Levy, & Gibson, 2020; Temperley & Gildea, 2018). Dependency length minimization can be a result of storage cost minimization, since storing a prediction for a longer time results in a larger storage cost when the cost of the entire sentence is evaluated. And we did observe significant effect of DLT storage cost on reading times. Whether CLT (integration or storage) cost provide better or supplementary explanation for word order variations remains as a future topic.

### The Importance of Storage Cost

With the current dataset, we observed that storage cost has a robust predictive power that is larger than that of (CLT) integration cost. This highlights the importance of storage cost in sentence processing, which is in fact a very old idea (Gibson, 2000; Just & Carpenter, 1992; Kimball, 1973; Lewis, 1996; Miller & Chomsky, 1963; Stabler, 1994; Yngve, 1960), though more recent, corpus-based reading times studies often exclusively focused on integration cost (Demberg & Keller, 2008; Dotlačil, 2021; Shain et al., 2016).

While DLT and CLT basically agree where storage cost should be observed, especially with regard to head-final structure, we argue that CLT storage cost is conceptually more straightforward than DLT storage cost. In CCG-based processing, categories like *S*/*NP* simultaneously represent a summary of the past inputs and predictions for future inputs. CLT simply posits that these CCG categories are the memory units. Then it naturally follows that cognitive costs of integration and storage are defined in terms of this unit. And it makes good predictions of reading times. In DLT, integration involves two heads in a dependency, but storage only counts one of them, the one that appears later. Dependency grammar does not explain why this has to be the case.

### Potential Extension to Parsing Preferences

Memory limitations can affect parsing preferences as well as reading times. While the current study focuses on increase in reading times due to CLT costs, a possible extension is to explain parsing preference by avoidance of CLT costs, as Gibson (2000) did with DLT, and as suggested by a reviewer. With regard to Japanese, there is a line of study investigating the attachment ambiguity of arguments to verbs (Aoshima et al., 2004; Kamide & Mitchell, 1999; Miyamoto, 2002; Nakano et al., 2002; Omaki et al., 2014). Kamide and Mitchell (1999) investigated sentences like (19), in which the dative noun *gakusei-ni* ‘student-dat’ is ambiguous between the matrix clause and embedded clause readings.

(19) Kyooju-ga  [_?_gakusei-ni [_?_tosyokansisyo-ga kasita] mezurasii komonjo-o
    professor-nom student-dat librarian-nom  lent unusual ancient.manuscript-acc
    miseta.
    showed
    ‘The professor showed (the student) the unusual ancient manuscript that the librarian had lent (the student).’

Kamide and Mitchell observed that Japanese speakers prefer the matrix reading, and took that result as supporting the pre-head hypothesis that structural analysis of arguments begins before the head (verb) is observed.

In CLT, the preference can be explained by the difference in the storage cost between the matrix and embedded readings when the ambiguous dative noun *gakusei-ni* is read. The matrix reading is simply obtained by clustering the dative noun with the preceding nominative, as in (20a). The storage cost at the dative noun is 1. In contrast, the embedded reading requires the parser to refrain from clustering at this point, and combining the dative noun with the following embedded subject, as in (20b). Note that we assume that the dative noun and the embedded subject are combined by the >**D**_x_ rule (Hoyt & Baldridge, 2008) to generate the prediction for a verb with the usual argument structure, (*T*\*NP*_*nom*_)\*NP*_*dat*_. The storage cost at the dative noun will be 2 in this case. If a parser resolves temporal structural ambiguity by minimizing storage cost, the matrix reading should be preferred.

(20) 
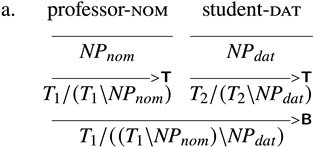
    
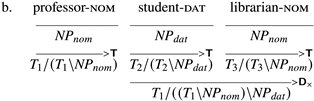

This line of research, however, also reveals a constraint imposed on psycholinguistic research using CLT. Aoshima et al. (2004) studied a more complicated case of temporal ambiguity regarding the attachment of a fronted *wh*-phrase (note that Japanese *wh*-phrases are in situ by default). They found that readers associate the *wh*-phrase with the embedded verb. They suggest that their result is due to the parser’s preference to assign a thematic role to the *wh*-phrase as early as possible. The result, however, contrasts with the aforementioned result of Kamide and Mitchell (1999), who found the preference for the matrix attachment. Aoshima et al. note that two factors of their materials may be responsible for the disagreeing results: even the matrix reading involves scrambling, and the ambiguous noun is a *wh*-element. To implement this account in CLT, we first have to formulate a CCG analysis of (long-distance) scrambled *wh*-phrase in Japanese. Analysis of *wh*-elements in Japanese is complicated by the fact that the scope of the question is marked by a particle *ka* attached to the verb, not by the *wh*-phrase as in English. Establishing such an analysis requires a thorough discussion in the realm of theoretical linguistics and is beyond the scope of this paper (in the current corpus study, we tentatively ignored the distinction between *wh*- and non-*wh*-phrases). This is an instance of the general constraint in using CLT that we need a theoretical analysis of the target phenomenon in CCG. Obtaining such an analysis is more difficult than drawing a dependency graph to generate predictions in DLT, because CCG has a more rigorous view on syntax and its relation to semantics. This situation is frustrating, but we believe it is something that linguistics must go through: if competence grammar and performance are closely related (and they should; see below), we should seek an explanation that addresses both theoretical and psycholinguistic questions.

### Parser-Grammar Relations

The current result shows that memory units defined by the limitation of the grammatical operations of CCG predict processing difficulty in real-time performance. This is in line with the strict competence hypothesis (SCH) (Steedman, 2000), according to which the parser does not build structure beyond what is defined by the competence grammar (Isono, 2024). SCH is the extreme version of the competence hypothesis, which assumes a close relationship between competence and performance (Ades & Steedman, 1982; Bresnan & Kaplan, 1982; Chomsky, 1965; Momma & Phillips, 2018; Phillips, 1996, 2012; Sag & Wasow, 2011). It should not be taken as collapsing the distinction between competence and performance, as competence-backed performance can still be limited by external factors such as memory limitations, attention, etc. Yet, SCH achieves a parsimonious account of human language faculty.

SCH can be understood by comparing CCG-based parsing with the left-corner parsing strategy. CCG-based parsing assumed here resembles arc-eager left-corner (AELC) parsing of a context-free grammar (CFG) (Resnik, 1992). For example, the CFG tree that an AELC parser would posit when the input is *John attacked* is as follows:

(21) 
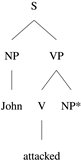

The analysis expressed by the tree is that the string *John attacked* will be an S if an NP follows. This is exactly what the CCG analysis expresses by assigning the category *S*/*NP* to the string. In fact, some studies using AELC parsing employ a CCG-like notation where a state like (21) is in fact expressed as *S*/*NP* (Rasmussen & Schuler, 2018; van Schijndel et al., 2013). AELC parsing has been popular in psycholinguistics since it correctly predicts the processing difficulty of center-embedded structures compared to left- and right-branching structures (Abney & Johnson, 1991). CCG-based (bottom-up) parsing provide an essentially same account, namely by the exceptionally large amount of memory space required. In CCG-based parsing (with canonical rules), there are three separate constituents when the parser reaches the depth of a doubly center-embedded structure:

(22) 
  $$\frac{\text{The}\;\text{reporter}\;\text{who}}{NP/\left(S/NP\right)}\;\frac{\text{the}\;\text{senator}\;\text{who}}{NP/\left(S/NP\right)}\;\frac{\text{John}}{NP}$$

Despite these similarities, CCG-based parsing complies with SCH, while left-corner parsing does not, and as a consequence, CCG-based parsing offers a simpler explanation of our incremental parsing ability (Isono, 2024). First, CCG-based parsing needs no special mechanism to handle displacement phenomena such as relativization since the grammar handles displacement and prediction with the same rules (**B** and **T**) that are used to model incrementality. Extending AELC to embrace displacement requires highly complicated extragrammatical structure building rules to ensure both incrementality and grammaticality, as seen in attempts to do that in Minimalist Grammars (Hunter, 2019; Stanojević & Stabler, 2018). Second, CCG-based parsing offers the semantics for the fragment for free, as it is defined in the grammar. This is crucial for a comprehension model because experimental facts show that the semantics of the fragment is available in real-time (Kamide et al., 2003; Tanenhaus et al., 1995). In AELC parsing, additional specification is needed for the semantics for the fragment.

## CONCLUSION

Characterizing memory-demanding sentences in a broad-coverage manner is important because it informs us about the structure and the structure-building algorithm that humans use in real-time sentence comprehension. We found that incremental parses offered by Combinatory Categorial Grammar (CCG) are able to characterize memory-demanding structures in Japanese. Taken together with the previous result in English, this suggests that memory-constrained, CCG-based processing is a promising theory of the universal mechanism underlying human sentence comprehension. According to this view, sentence comprehension proceeds as logical inference that efficiently summarizes the previous input into a few categories and constantly updates them under a tight memory constraint.

## ACKNOWLEDGMENTS

We thank Edward Gibson, Yuki Hirose, Kentaro Nakatani, Douglas Roland, and participants of the 168th Annual Meeting of the Linguistic Society of Japan for valuable comments to earlier versions of this work.

## FUNDING INFORMATION

This work is supported by JSPS KAKENHI Grant numbers JP23KJ0538 and JP25K22996, and the NINJAL research project.

## DATA AND CODE AVAILABILITY STATEMENTS

The BCCWJ corpus is available at https://clrd.ninjal.ac.jp/bccwj/en/index.html (license required). The UD annotation of the corpus is available at https://universaldependencies.org/treebanks/ja_bccwj/index.html. The BCCWJ-SPR2 dataset is available at https://github.com/masayu-a/BCCWJ-SPR2.

The code for reproducing the current analysis using these data (calculation of the predictors and the statistical analysis) is available at https://osf.io/62utg/.

## ETHICS APPROVAL

This study does not involve any acquisition of new data from human participants that would require ethics approval.

## Notes

^1^ There are two different notational conventions for functional categories. The one we adopt here, used in CCG, always places the resulting category to the left of the argument category. The other, used in type-logical variants (Lambek, 1958, 1961, i.a.) places the resulting category always on top of the argument category.^2^ Steedman and colleagues have assumed that type-raising only takes place in the lexicon and applies only to certain categories (Steedman, 2026; Steedman & Baldridge, 2011). One reason for this is that type-raising of some categories, such as *N* and *S*\*NP*, overgenerates island-violating sentences. As Isono (2024) points out, however, such a restriction is too strong and undergenerates. This study follows Isono (2024) in assuming type-raising is a syntactic operation and some other factors, not necessarily syntactic, are responsible for the island phenomena (Chaves & Putnam, 2020; Kubota & Levine, 2020)^3^ The dependency tree assumed here, based on Universal Dependencies, is different from that assumed in the original DLT paper (Gibson, 2000) in that there is no trace. This does not change the prediction that object relative clauses are costly at the embedded verb compared to subject relative clauses.^4^ https://universaldependencies.org/treebanks/ja_bccwjluw/.^5^ Two versions of UD annotation for BCCWJ are published depending on the granularity of the morphological analysis. We use the coarser version (the *long unit word* version; Omura et al., 2021) since DLT and CLT are mainly concerned with syntactic structures.^6^ As a reviewer pointed out, another rule that is often used in CCG and could be included in the list is the forward crossed composition rule >**B**_x_ (Steedman, 2000). It can be used in Japanese when the order of the dependents does not follow the canonical order specified by the head (e.g., scrambling). If that line of analysis is employed, the CLT cost will increase (if we just employ the >**B**_x_ rule) compared to the canonical order, or stay the same (if we also employ another rule that enables incremental processing of scrambled word order). In this study, we adopt a simplifying assumption that all the observed word orders are canonical and avoid using the >**B**_x_ rule. This means that we focus on the costs due to where the dependents are placed relative to each other, but not the potential cost of scrambling.^7^ The sentence ID given in the UD annotation is PB32_00008-50; the final verb cluster *kaki-okut-ta-nodeat-ta* ‘write-send-past-emphasis-past’ is simplified for the reason of space. The most incremental CCG tree (without the >**B**^2^ rule) for the sentence is as follows:

(i) 
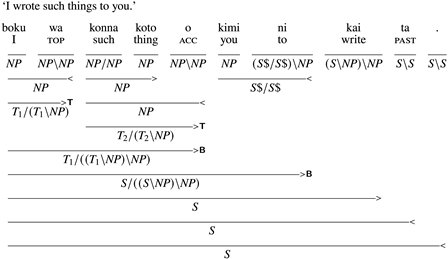

The $ symbol in the category of *ni* ‘to’ indicates an arbitrary number of arguments. See Steedman (2000) for the definition of the symbol, and Bekki (2010) for the use of the symbol for the analysis of Japanese adverbials.^8^ As a reviewer points out, GPT surprisal already exists as a powerful metric of predictability. Isono (2024) found, however, that dependency-based integration count is significant even when GPT surprisal is included in the regression model. This is possibly because human prediction relies more on structured representation of recent context, while GPT has access to much longer context (for a related observation, see Kuribayashi et al., 2022). For this reason, the current analysis also includes both integration count and GPT surprisal.^9^ To address the issue of zero-frequency morpheme in the frequency table, we adopted a smoothing approach by assigning a frequency of 1 to morphemes with zero frequency. The frequency table URL: https://www.gsk.or.jp/catalog/gsk2020-c/.^10^ https://huggingface.co/rinna/japanese-gpt2-xsmall.^11^ The largest model (DLT + CLT model) is specified as follows in the R syntax.

(i) log(SPR_reading_time) ∼ SPR_sentence_ID + SPR_bunsetsu_ID+
    nchar + nchar_so + uni_surp + uni_surp_so + gpt_surp + gpt_surp_so+
    dlt_cnt + dlt_cnt_so + clt_cnt_Z + clt_cnt_Z_so+
    dlt_int_X + dlt_int_X_so + dlt_stor + dlt_stor_so+
    clt_int_Y_Z + clt_int_Y_Z_so + clt_stor_Z + clt_stor_Z_so

X, Y, and Z are variables that specify the variant of the theory, namely distance metric (X, Y ∈ {nv, nvjr, char}) and rule set (Z ∈ {B1, B2}). _so stands for spillover, i.e., the value from the previous region. Other models simply lack some of the terms included here.^12^ The tests here are susceptible to false positives, since we have no prior hypotheses about which POS tag may be predictive of Δ*LL* and yet adopt *α* = 0.05 for each POS tag. This is fine because the purpose of this test is to find POS tags to be included in the baseline model for further testing. We do not draw any conclusion from the tests here alone.

## Supplementary Material


