# Supplementary material for: Modeling Memory Effects in a Head-Final Language With Category Locality
Source: Open Mind (Camb). 2026 Aug 21;10:1359–93. doi: 10.1162/OPMI.a.384 (PMC13618756; doi:10.1162/OPMI.a.384)
Supplement: Supplementary file 1 [file opmi-10-1359-s001.pdf]

Appendices to: Isono, S., Kajikawa, K., Oseki, Y., & Asahara, M. (2026). Modeling Memory Effects in a Head-Final Language with Category Locality. *Open Mind: Discoveries in Cognitive Science.*

Appendix I: UD to CCG conversion

This appendix describes the conversion method used to obtain the most incremental CCG parse from the UD annotation for the sentences in the dataset. I will use as an example the sentence in Figure 3 of the main text, which is a simplified version of an actual sentence in the corpus.

Based on the UD annotation that is given to the actual sentence, we will assume the following UD annotation for our example sentence:

(1)
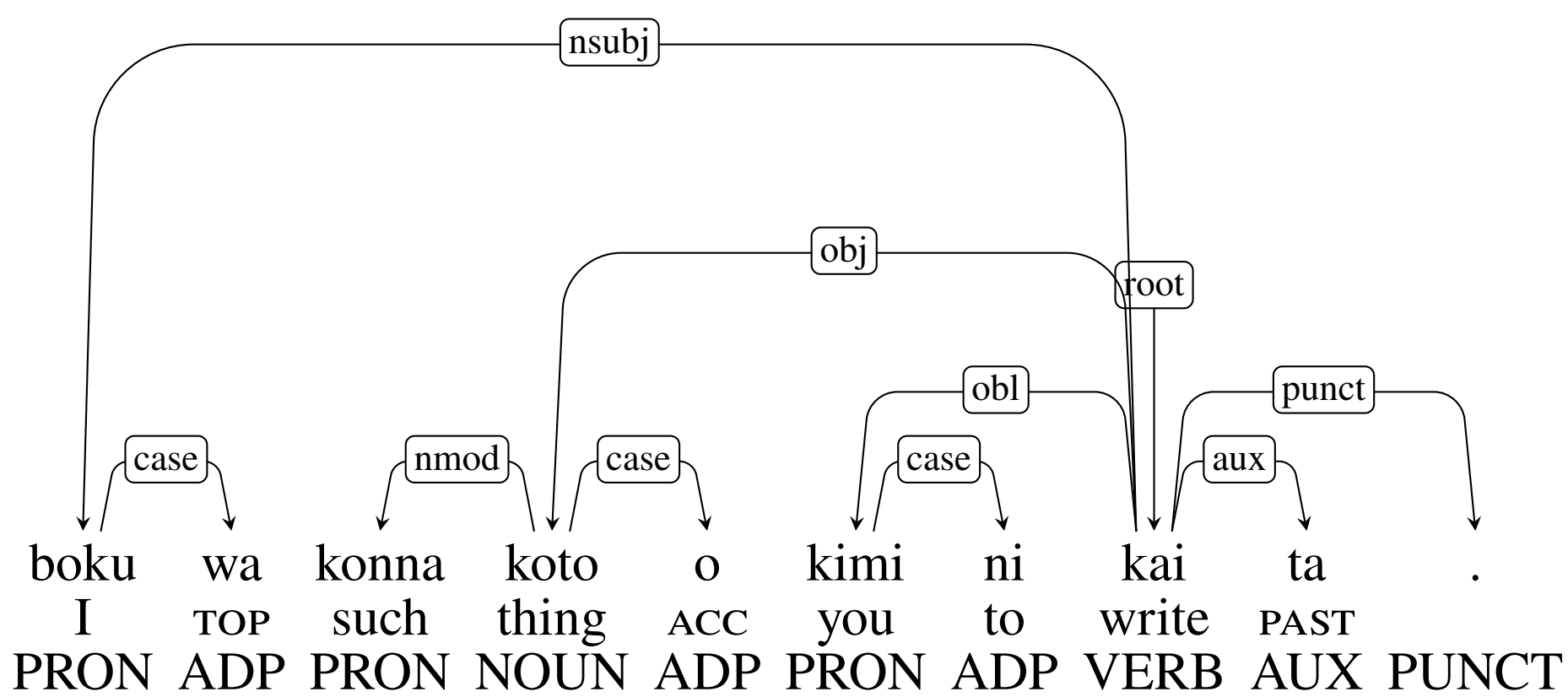

First, any crossed dependencies were fixed so that they don't cross, since they don't translate to a constituent structure straightforwardly. If the dependencies (h_1, d_1) and (h_2, d_2) cross, h_1, h_2 being the heads and d_1, d_2 being the dependents, and h_2 is dominated by h_1 (i.e., tracing the dependencies upwards from h_2 leads to h_1), then the dependency (h_2, d_2) was replaced by (h_1, d_2). This process affected 161 sentences (1.7%) in the dataset.

The dependency structure was then converted to a tree structure. This is achieved by translating each dependency to a binary-branching structure. When there are multiple dependencies that share the head, the rightward furthest dependency is targeted first, followed by closer rightward dependencies; after all the rightward dependencies are resolved, the leftward

furthest dependency is targeted, followed by closer leftward dependencies. This process generates the following tree structure for (1):

(2)

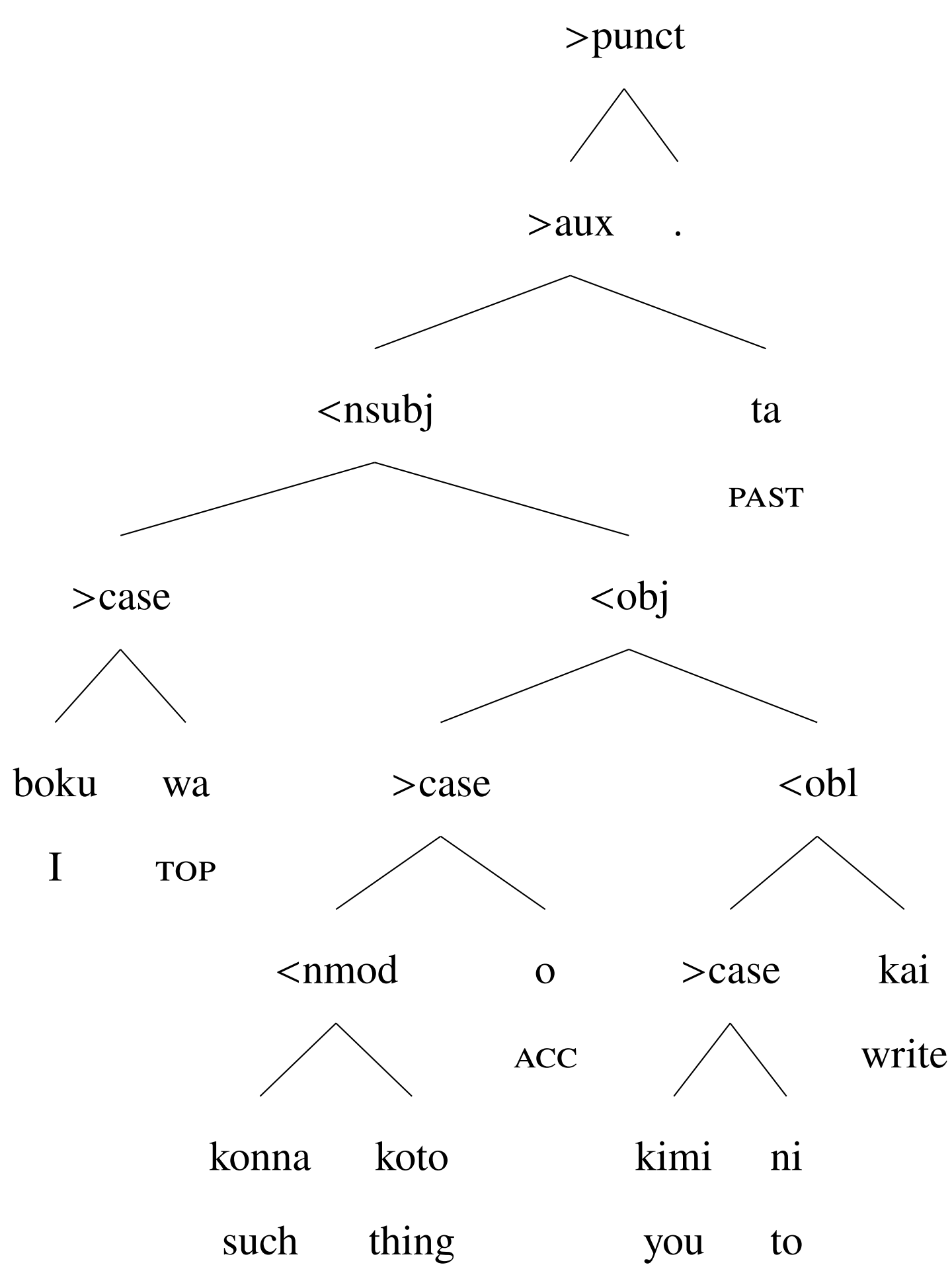

Each non-terminal node is labeled with the type and the direction of the dependency (the latter is indicated by > and <). The direction of the CCG application at each non-terminal node is determined based on these pieces of information. If the dependency type is an argument-head dependency such as `nsubj`, the dependency direction matches the CCG application direction. The direction must be reversed in the following two cases. The first case is when the dependent is an adjunct, as in the case of `obl` and `nmod` dependencies here. While in dependency grammar adjuncts are dependent on the modificand, in CCG adjuncts of category X/X select the modificand of category X. This also applies to `punct` dependencies since punctuations can be

seen as adjuncts. The second case is where CCG adopts a different view regarding which element is the head. This applies to `case`, and `aux`. CCG analyses of languages with morphological case markers assume that case markers select the noun (Bekki, 2010; Bozsahin, 2002; Steedman, 2019). Auxiliaries are standardly assumed to select the verb (Steedman, 2000, 2019, 2023), as in Minimalist syntax. Mapping the direction of dependencies to the direction of applications under these considerations yields the following tree:

(3)

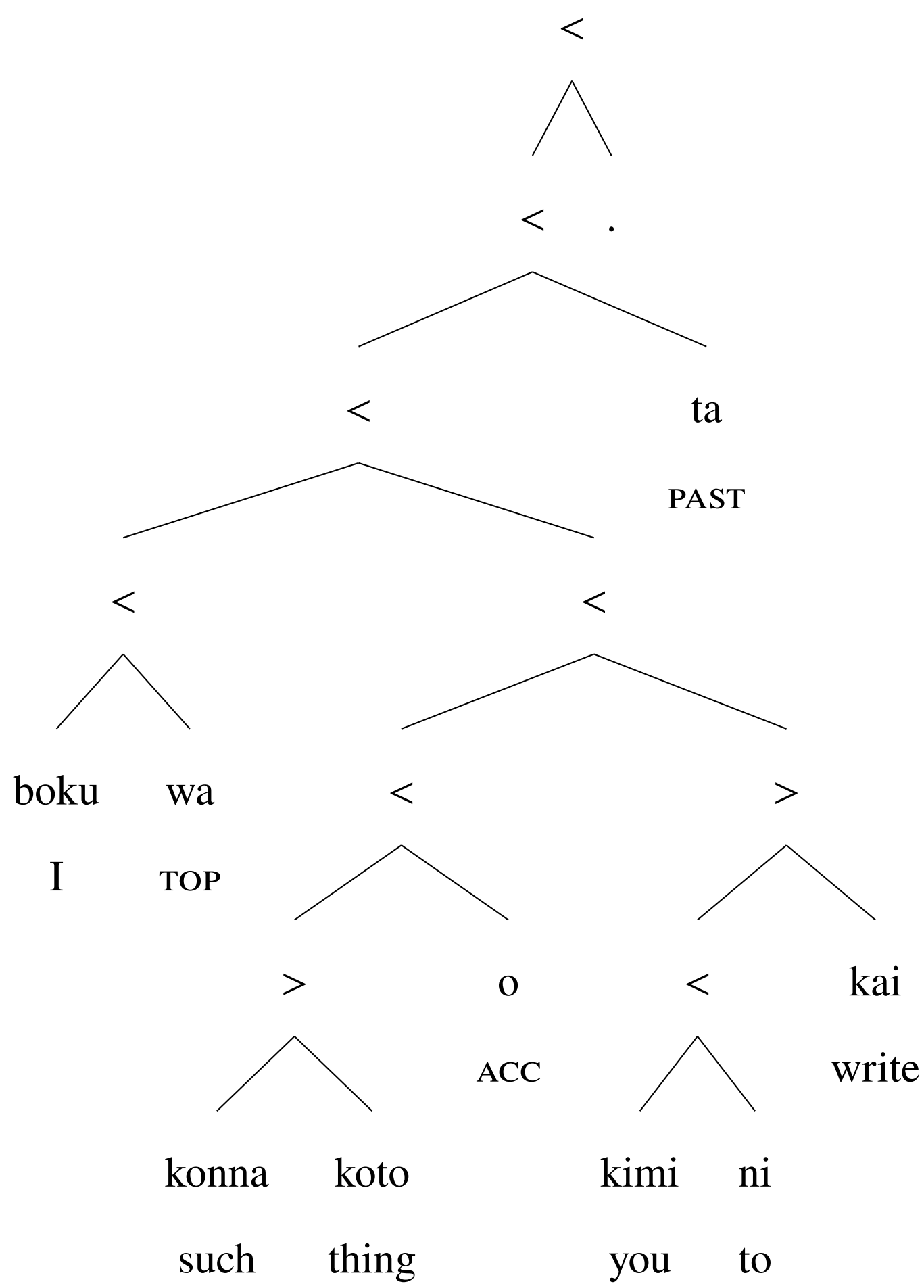

Or, in the standard proof tree style:

(4)

boku	wa	konna	koto	o	kimi	ni	kai	ta	.
I	TOP	such	thing	ACC	you	to	write	PAST	
A	$B\backslash A$	C/D	D	$E\backslash C$	F	$(((G\backslash B)\backslash E)/H)\backslash F$	H	$I\backslash G$	$J\backslash I$

$A \;\; B\backslash A \Rightarrow_{<} B$; $C/D \;\; D \Rightarrow_{>} C$; $F \;\; (((G\backslash B)\backslash E)/H)\backslash F \Rightarrow_{<} ((G\backslash B)\backslash E)/H$

$C \;\; E\backslash C \Rightarrow_{<} E$; $((G\backslash B)\backslash E)/H \;\; H \Rightarrow_{>} (G\backslash B)\backslash E$

$E \;\; (G\backslash B)\backslash E \Rightarrow_{<} G\backslash B$

$B \;\; G\backslash B \Rightarrow_{<} G$

$G \;\; I\backslash G \Rightarrow_{<} I$

$I \;\; J\backslash I \Rightarrow_{<} J$

This conversion process does not specify the actual categories of the constituents such as NP and S. The abstract shape of the category can however be inferred from the rules, as indicated in the above tree with variable categories $A, B, \ldots,$ and that is sufficient to calculate the CLT predictors because the predictors are defined over the abstract shape of the tree. The actual categories can be obtained by replacing variable categories ($A \rightarrow NP$, $H \rightarrow (S\backslash NP)\backslash NP$, etc.), if one needs them.

The CCG tree thus obtained is in the Eisner normal form (Eisner, 1996). It is then converted to the most incremental tree by rotating right-branching trees to left-branching ones. The search starts from the top subtree, and whenever a right-branching tree with an appropriate combination of rules is found, it is immediately converted to the left-branching tree. For example, a subtree of (4) is as follows:

(5) $B \quad E \quad (G\backslash B)\backslash E$

$E \;\; (G\backslash B)\backslash E \Rightarrow_{<} G\backslash B$

$B \;\; G\backslash B \Rightarrow_{<} G$

This is a right-branching tree with two backward applications (<). It can be converted to the following left-branching tree.

(6)

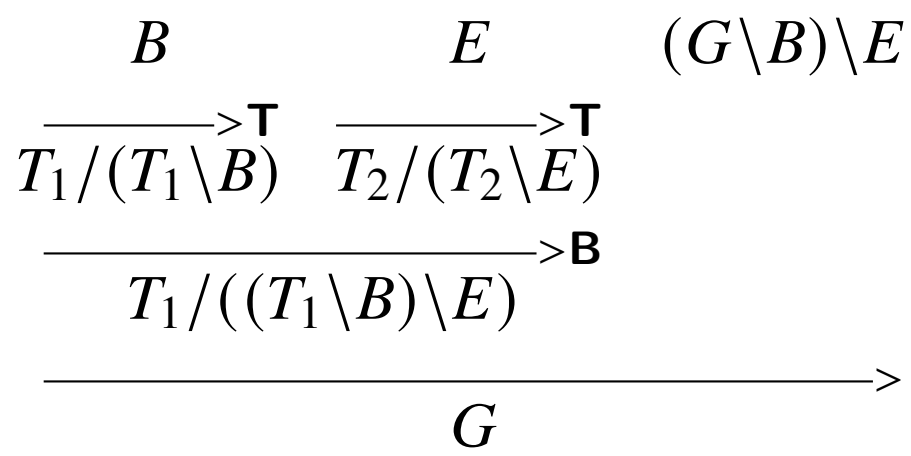

By recursively applying such conversions, we obtain the following most incremental tree:

(7)

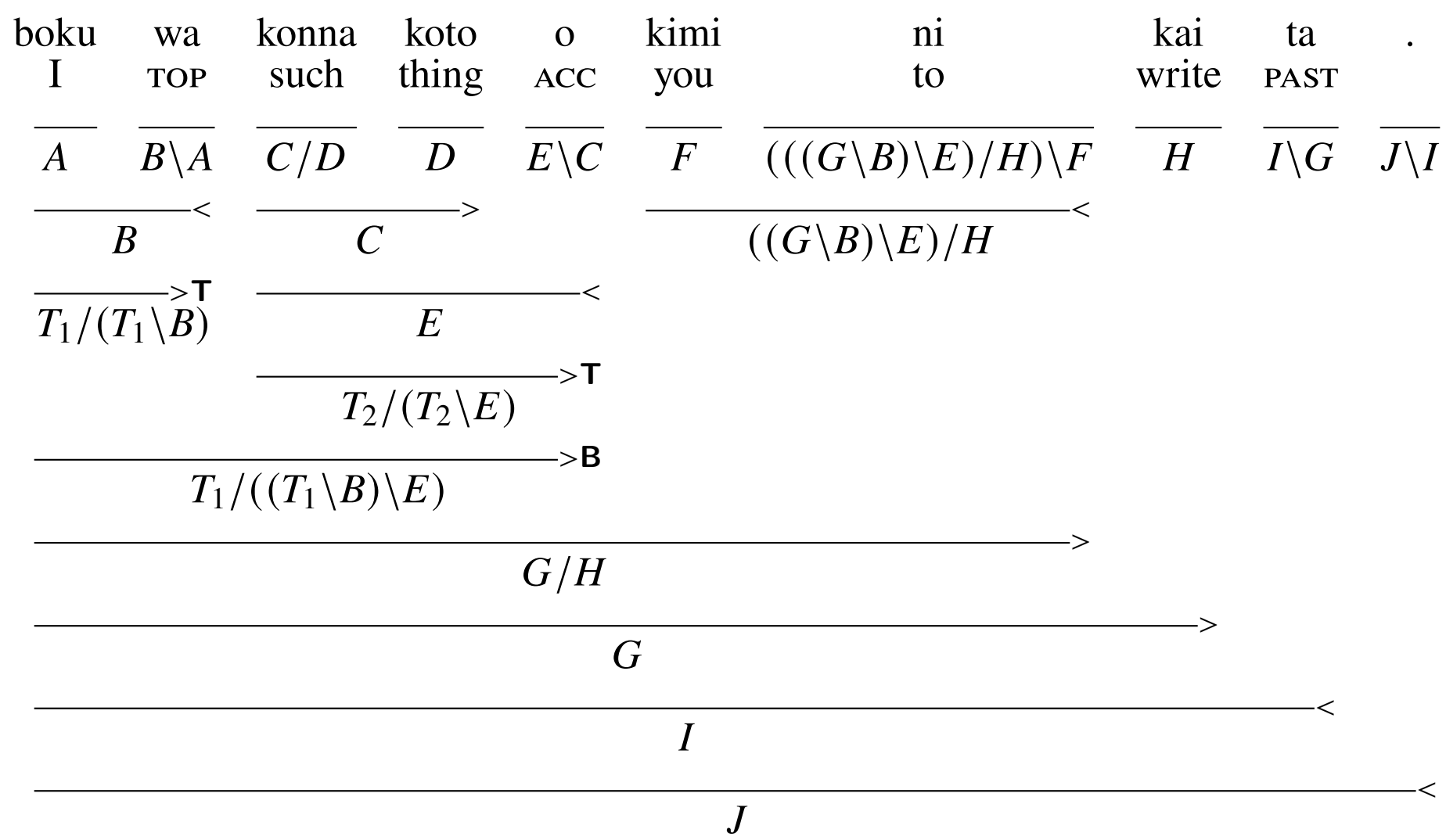

The CLT predictors are calculated from this tree.

Appendix II: Initial analysis using linear mixed-effects modeling

As described in the main text, we initially analyzed the reading time data by fitting linear mixed-effects models to the entire data, following some previous studies (Demberg & Keller, 2008; Dotlačil, 2021; Isono, 2024). We faced several problems in the analysis and switched to another approach reported in the main text. In this Appendix, we report the results of the initial analysis.

Methods

As in the final analysis, the dataset was split into the exploratory and confirmatory dataset. The best-performing variants of DLT and CLT in the exploratory stage, identified by log likelihoods, were used in the confirmatory stage. In each stage, the entire data was modeled by linear mixed-effects models (Baayen et al., 2008) with or without DLT and CLT costs. The

		NV	NVJR	Char
DLT		−2	**17**	−3
CLT	without $>\mathbf{B}^2$	34	5	**42**
	with $>\mathbf{B}^2$	−8	−25	12

Table 1

Delta log likelihoods of the models considered in the exploratory analysis. The best-performing models (with the highest log likelihood) are highlighted with bold. NV: 1 distance unit per noun and verb; NVJR: 1 distance unit per noun, verb, adjective, and adverb; Char: 1 distance unit per character.

formulas were identical to the ones used in the final analysis except that they also contained random intercepts for samples and participants. Random slopes were not included although it would be desirable to do so to ensure the generalizability of the significant fixed effects (Barr et al., 2013). Unfortunately, the maximal model with all the possible by-participant random slopes resulted in a singular fit. As Barr et al. (2013) recommend keeping random slopes for variables of theoretical interest, we then tried a model with random slopes for DLT and CLT predictors, but this model also resulted in a singular fit. We thus ended up using models without random slopes, but the results must be interpreted with caution.

Results

Table 1 summarizes the performance of the models considered in the exploratory analysis. For the ease of interpretation, the performance is indicated by the increase ΔLL compared to the baseline model with just the control predictors. The best-performing DLT model was the one that used the number of content words as the distance metric. The best-performing CLT model was the one that used the number of characters as the distance metric and was without the $>\mathbf{B}^2$ rule.

With the best-performing variants of DLT and CLT thus identified, four models were fitted in the confirmation stage: the baseline model with control predictors only, the DLT model, the CLT model, and the DLT+CLT model. Table 2 summarizes these models. Likelihood ratio tests

showed that both DLT and CLT models performed better than the baseline model ($ps < 0.001$), and the DLT+CLT model performed better than the DLT and CLT models ($ps < 0.001$). ΔLL of the DLT model was larger than ΔLL of the CLT model.

A difficult aspect of this result is the sign of the coefficients. While CLT integration cost showed positive (slowdown) effects, as expected, DLT integration cost showed negative (speedup) effects, and DLT/CLT storage costs showed mixed signs. This could be due to overfitting, multicollinearity, or some genuine linguistic effect. To eliminate the possibility of overfitting, we switched to the evaluation of predictive fit, reported in the main text. We also inspected for multicollinearity because it can result in instable coefficient estimates (Friedman & Wall, 2005). We inspected multicollinearity with the `check_collinearity` function from the `performance` package in R (Lüdecke et al., 2021). The results suggest that multicollinearlity may have affected some of the estimates (see Table 2).

	Baseline model		DLT model			CLT model			DLT+CLT model		
			ΔLL = 386			ΔLL = 118			ΔLL = 629		
Intercept	5.7323	***	5.7323	***		5.7324	***		5.7300	***	
Position of sentence	-0.0384	***	-0.0385	***		-0.0385	***		-0.0385	***	
Position of bunsetsu	-0.0045	***	-0.0032	***		-0.0047	***		-0.0038	***	
No. of characters	0.0134	***	0.0139	***		0.0132	***		0.0137	***	
(Spillover)	-0.0002		0.0000			-0.0003			-0.0002		
Unigram surprisal	0.0437	***	0.0442	***		0.0441	***		0.0442	***	
(Spillover)	0.0378	***	0.0377	***		0.0380	***		0.0381	***	
GPT surprisal	-0.0007	**	-0.0008	**		-0.0007	**		-0.0008	**	
(Spillover)	0.0058	***	0.0059	***		0.0059	***		0.0060	***	
DLT integration count	-0.0099	***	-0.0030	***	!	-0.0093	***		-0.0015	***	!
(Spillover)	-0.0078	***	-0.0039	***	!	-0.0072	***		-0.0023	***	!
CLT integration count	-0.0061	***	-0.0123	***	!	-0.0076	***	!	-0.0134	***	!
(Spillover)	-0.0099	***	-0.0118	***	!	-0.0112	***	!	-0.0141	***	!
DLT integration cost			-0.0075	***					-0.0090	***	
(Spillover)			-0.0056	***					-0.0066	***	
DLT storage cost			-0.0037	***					-0.0041	**	!!
(Spillover)			0.0013	***					0.0144	***	!!
CLT integration cost						0.0018	***		0.0025	***	
(Spillover)						0.0010	***		0.0024	***	
CLT storage cost						-0.0004			0.0016		!!
(Spillover)						-0.0008	**		-0.0117	***	!!

Table 2

*Estimates of four LME models in the confirmation stage. Significance codes: *** for* $p < 0.001$*, ** for* $p < 0.01$*, * for* $p < 0.05$*. Multicollinearity codes: !! for* $10 \leq \text{VIF}$ *and ! for* $5 \leq \text{VIF}$

References

Baayen, R. H., Davidson, D. J., & Bates, D. M. (2008). Mixed-effects modeling with crossed random effects for subjects and items. *Journal of Memory and Language*, *59*(4), 390–412. https://doi.org/10.1016/j.jml.2007.12.005

Barr, D. J., Levy, R., Scheepers, C., & Tily, H. J. (2013). Random effects structure for confirmatory hypothesis testing: Keep it maximal. *Journal of Memory and Language*, *68*(3), 255–278. https://doi.org/10.1016/j.jml.2012.11.001

Bekki, D. (2010). *Nihongo bunpoo no keisiki riron [Formal theory of Japanese grammar]*. Kurosio.

Bozsahin, C. (2002). The combinatory morphemic lexicon. *Computational Linguistics*, *28*(2), 145–186. https://doi.org/10.1162/089120102760173634

Demberg, V., & Keller, F. (2008). Data from eye-tracking corpora as evidence for theories of syntactic processing complexity. *Cognition*, *109*, 192–210.

Dotlačil, J. (2021). Parsing as a cue-based retrieval model. *Cognitive Science*, *45*(e13020). https://doi.org/10.1111/cogs.13020

Eisner, J. (1996). Efficient normal-form parsing for Combinatory Categorial Grammar. *34th Annual Meeting of the Association for Computational Linguistics*, 79–86. https://doi.org/10.3115/981863.981874

Friedman, L., & Wall, M. (2005). Graphical views of suppression and multicollinearity in multiple linear regression. *The American Statistician*, *59*(2), 127–136.

Isono, S. (2024). Category Locality Theory: A unified account of locality effects in sentence comprehension. *Cognition*, *247*, 105766. https://doi.org/10.1016/j.cognition.2024.105766

Lüdecke, D., Ben-Shachar, M. S., Patil, I., Waggoner, P., & Makowski, D. (2021). performance: An R package for assessment, comparison and testing of statistical models. *Journal of Open Source Software*, *6*(60), 3139. https://doi.org/10.21105/joss.03139

Steedman, M. (2000). *The syntactic process*. MIT Press. https://doi.org/10.7551/mitpress/6591.001.0001

Steedman, M. (2019). Combinatory Categorial Grammar. In A. Kertész, E. Moravcsik, & C. Rákosi (Eds.), *Current approaches to syntax: A comparative handbook* (pp. 389–420). De Gruyter Mouton. https://doi.org/10.1515/9783110540253-014

Steedman, M. (2023). On internal merge. *Linguitic Inquiry*, *Advance publication*. https://doi.org/10.1162/ling_a_00521
